# A bioinformatic approach to characterize the vitellogenin receptor and the low density lipoprotein receptor superfamily in the newt *Cynops orientalis*

**DOI:** 10.1038/s41598-025-88011-6

**Published:** 2025-01-27

**Authors:** Chiara Spinsante, Federica Carducci, Elisa Carotti, Adriana Canapa, Davide Bizzaro, Maria Assunta Biscotti, Marco Barucca

**Affiliations:** Department of Life and Environmental Sciences, Polytechnic University of Marche, Via Brecce Bianche, 60131 Ancona, Italy

**Keywords:** Amphibians, Vitellogenin receptor, Basal sarcopterygians, Low density lipoprotein receptors gene family, LDLR, VTGR, Evolutionary biology, Gene expression

## Abstract

The Low Density Lipoprotein receptors (LDLRs) gene family includes 15 receptors: very low-density lipoprotein receptor (VLDLR), LDLR, Sorting-related receptor with A-type repeats (SORLA), and 12 LDL receptor-related proteins (LRPs): LRP1, LRP1B, LRP2, LRP3, LRP4, LRP5, LRP6, LRP8, LRP10, LRP11, LRP12, LRP13. Most of these are involved in the transduction of key signals during embryonic development and in the regulation of cholesterol homeostasis. In oviparous animals, the VLDL receptor is also known as VTGR since it facilitates the uptake of vitellogenin in ovary. In tetrapods, information concerning genes encoding these proteins is limited to a few taxa. Here, we report the characterization of VTGR in the amphibian *Cynops orientalis*. The secondary structure analyses and the expression profiles obtained from hepatic and gonadal tissues of *C. orientalis* supported the role of VTGR as vitellogenin oocyte membrane receptor in this species. Moreover, to get a holistic view of the evolutionary history of this gene superfamily, we extended our investigation to all 15 genes belonging to the LDLR superfamily analyzing through a phylogenetic analysis a total of 161 sequences belonging to 11 genera of vertebrates. The position of LRP8 in the tree and its expression findings in *C. orientalis* ovary allowed us to suggest that other proteins of the LDLR superfamily could act as receptors during vitellogenesis.

**Supplementary Information:**

The online version contains supplementary material available at 10.1038/s41598-025-88011-6.

## Introduction

In non-mammalian vertebrates, vitellogenin (Vtg) is the precursor of the egg yolk, that is the main nutritional reserve for embryo development^1^. This multidomain apolipoprotein is synthetized in female liver of oviparous and ovoviviparous species during reproductive season^2^. Low levels of *vtg* have been recorded also in males^2–5^. Additional Vtg functions have been attributed as in egg buoyancy^6,7^, in host defense^8–11^, and toxin transport^12^.

**Fig. 1:**
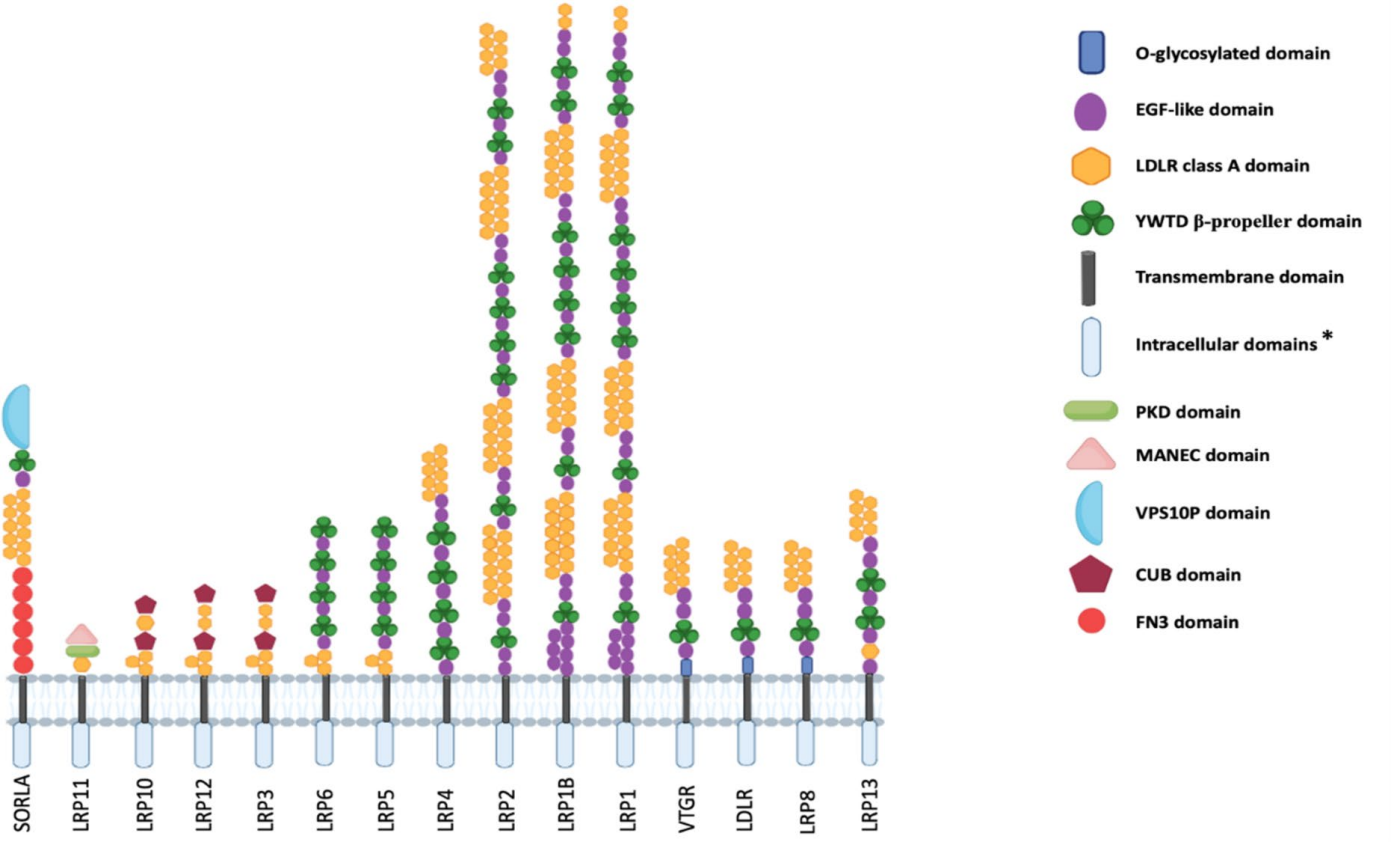
Domains organization of the LDLR superfamily members. Domains of the low density lipoprotein receptor (LDLR) superfamily members are showed. They are membrane-anchored and contain a transmembrane, a cytoplasmic, and an extracellular domain. The represented domains are: Epidermal Growth Factor (EGF), LDLR class A domain (LDLRA), Tyr-Trp-Thr-Asp (YWTD) β-propeller, Polycystic Kidney Disease (PKD), N-terminus with Eight Cysteines (MANEC), Vacuolar Protein Sorting ten Protein (VPS10P), complement C1r/C1s, Uegf, Bmp1 (CUB), and fibronectin type-III (FN3). Very Low Density Lipoprotein Receptor (VLDLR), LDLR, and Low-Density Lipoprotein Receptor-Related Protein 8 (LRP8) comprise an additional O-glycosylated extracellular domain adjacent to the transmembrane domain. The domain description refers to Principe et al. (2021)^24^. *Asterisk means that for the intracellular domain are not representative of the real length.

In vertebrates, in which multiple *vtg* genes are present^1,5,13–19^, the Vtg production is regulated by hypothalamic–pituitary–gonadal (HPG) axis that leads to 17β-estradiol release (E2) by follicular cells. This hormone is recognized by estrogen receptors located in hepatocytes stimulating the synthesis of *vtg* genes^20^. After post-translational modifications, the produced proteins are released in the bloodstream and reach the oocyte in the form of a homodimeric complex where it is incorporated through Vtg receptor (VTGR)-mediated endocytosis. The VTGR, known also as very-low-density lipoprotein receptor (VLDLR)^21,22^, is member of the low-density lipoprotein receptor (LDLR) superfamily that includes also LDLR, LRP1, LRP1B, LRP2, LRP3, LRP4, LRP5, LRP6, LRP8, LRP10, LRP11, LRP12, LRP13 and the Sortilin-related receptor with A-type repeats (SORLA)^23,24^. They are type 1 transmembrane receptors having an extracellular domain made up of cysteine-rich ligand-binding repeats (LBRs) responsible for the ligand-binding activity, a transmembrane domain, and an intracellular domain. Differently from the extracellular domain, the cytoplasmic domain is less conserved except for Asn-Pro-X-Tyr (NPxY) motif involved in adapter protein docking and present in VLDLR, LDLR, LRP8, LRP4, LRP1, LRP1B, LRP2, and SORLA. Moreover, VLDLR, LDLR, and LRP8 show an O-glycosylated domain close to the transmembrane domain. These three proteins share a similar domain organization. However, LRP8 and LDLR present seven LDLR class A domains while VLDLR has eight of these domains^25^ (Fig. 1). LRP8 and LDLR differ for the length of intracellular domain. Although the members of the LDLR family are primarily involved in binding lipoproteins, other functions have been identified such as in neuronal development^26^, signaling transduction during embryo development^27^, and cholesterol homeostasis^28^. Moreover, the genotypization of some *ldlr* gene SNPs highlighted that these might be associated with a higher risk in the onset of Alzheimer disease, especially in human female individuals^29^.

The binding site of Vtg for VTGR is in the N-terminal of the lipovitellin I domain. In particular, Li and colleagues (2003)^30^ have demonstrated that Lys185 located in the 84-amino acid fragment at the N-terminus of Vtg is important for electrostatic interaction with VTGR and that the first three LBRs of the receptor were found to be essential for this ionic interaction. Three-dimensional structure information has been provided by Daly and colleagues (1995)^31^ through two-dimensional NMR spectroscopy. In detail, authors have showed that the N-terminal cysteine-rich repeats are characterized by a beta-hairpin structure, followed by a series of beta-turns. In fish, besides VTGR, LRP13 has been designed as vitellogenin oocyte receptors^23,32–34^. In tetrapods, VTGRs and the LDLR superfamily have been investigated in a restricted number of taxa^35^. In detail, the process of Vtg/VTGR endocytosis has been investigated in oviparous amphibians^36^, while the binding of Vtg/VTGR has been described in the African clawed frog^37^ and in chicken^38^. In mammals, the VTGR is known as VLDLR and is widely expressed in the heart, in the skeletal muscle, and in the adipose tissue^28^.

Due to the paucity of information in amphibians, the aim of this work was to characterize VTGR in the newt *Cynops orientalis.* We extended our investigation to the other 14 genes belonging to the LDLR superfamily, screening the available transcriptomic data of *C. orientalis* and the genomic and transcriptomic resources of 10 other vertebrate species. A total of 161 sequences belonging to 11 genera was included in the phylogenetic analysis carried out to unravel the evolutionary history of the LDLR superfamily. Analysis of secondary structures coupled with the hepatic and gonadal expression profiles in *C. orientalis* supported its role as vitellogenin oocyte membrane receptor in this species. In addition, the position of LRP8 in the tree and its expression in *C. orientalis* ovary suggested that other members of this family could act as receptors during vitellogenesis.

## Results

### Identification of genes encoding low density lipoprotein receptors in *Cynops orientalis* and phylogenetic analysis

The sequence of VTGR was retrieved from the in-house *de novo* assembled transcriptome of *C. orientalis*. To validate its identity, the investigation was extended to the other 14 genes of the LDLR superfamily. In the newt, 12 sequences were retrieved from our high-quality transcriptome, ten of which were complete. To evaluate the identity and orthology of these sequences 10 other vertebrate species were considered (Supplementary Table S1). Regarding the two basal sarcopterygians, the coelacanth *L. menadoensis* and the lungfish *P. annectens*, in-house *de novo* assembled transcriptomes were screened to identify ortholog sequences. Eight sequences were retrieved for *L. menadoensis* (one of which labelled as incomplete), while the other seven sequences were obtained from the genome of its congeneric *L. chalumnae.* Concerning *P. annectens*, a total of nine complete sequences were obtained, while public repositories were screened for the remaining six sequences. Sequences related to the two actinopterygians and the other six tetrapods were downloaded from public repositories. Details about identification and characterization of sequences analyzed in this study were reported in Supplementary Table S1.

**Fig. 2:**
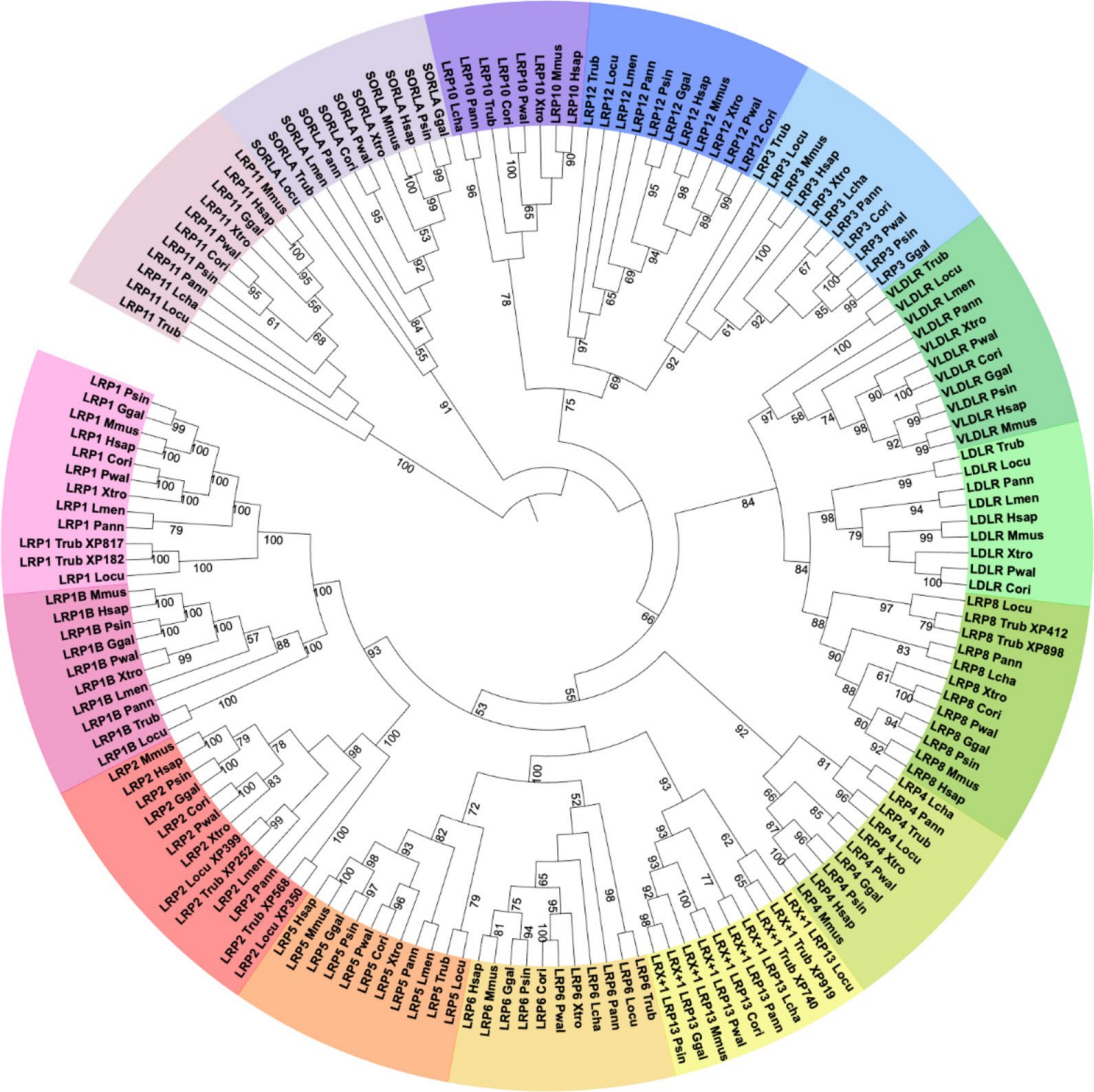
Maximum Likelihood phylogenetic reconstruction of receptors belonging to the LDLR superfamily. Circular representation of the Maximum Likelihood phylogenetic reconstruction performed on the other members belonging to the Low Density Lipoprotein Receptor (LDLR) superfamily, with bootstrap values. The different colors aimed to highlight the different receptors belonging to the superfamily. Locu: *Lepisosteus oculatus*, Trub: *Takifugu rubripes*, Lmen: *Latimeria menadoensis*, Lcha: *Latimeria chalumnae*, Pann: *Protopterus annectens*, Cori: *Cynops orientalis*, Pwal: *Pleurodeles waltl*, Xtro: *Xenopus tropicalis*, Psin: *Pelodiscus sinensis*, Ggal: *Gallus gallus*, Mmus: *Mus musculus*, Hsap: *Homo sapiens*.

A total of 161 sequences belonging to 11 genera representing the main evolutionary lineages of vertebrates were included in the phylogenetic analysis. The tree topology showed 15 clades corresponding to each member of the LDLR superfamily (Fig. 2). It was noteworthy that for each clade the position of the sequences reflects the evolutionary relationships of the analyzed species. In particular, within each clade, two monophyletic groups can be distinguished: that including actinopterygians (ray-finned fish, *L. oculatus* and *T. rubripes*) and that comprising sarcopterygians (lobe-finned fish and tetrapods). In the case of sarcopterygians, sequences related to *Latimeria* and *Protopterus* branched external to those of tetrapods. An exception was represented by the duplicate LRP2 sequences (XP_029693252.1 and XP_015214399.1) of *T. rubripes* and *L. oculatus*, that were placed within the group characterized by sarcopterygians, unlike the sequences XP_029693568.1 of *T. rubripes* and XP_015217350.1 of *L. oculatus* that were placed externally. This might be explained considering the so-called “long branch attraction phenomenon”. Indeed, the sequences of *L. menadoensis* and *P. annectens* were attracted by those of fish due to their high divergence compared to those of the other sarcopterygians. Besides *lrp2*, in *T. rubripes*, the presence of duplicate genes emerged for *lrp1*, *lrx + 1* (*lrp13*), and *lrp8*. In particular, the microsyntenic analyses revealed that the two copies of *lrp1*, *lrp8*, and *lrp13* in *T. rubripes* were located on separate chromosomes while *lrp2* copies were present on the same chromosome in the case of *T. rubripes* and on two distinct linkage groups in the spotted gar (see Supplementary File S1).

In general, the tree topology presented in the external position the sequences related to LRP11 and SORLA. Closer paralog relationships supported by high bootstrap values were highlighted for LRP10/LRP12/LRP3, VLDLR/LDLR/LRP8, and LRP2/LRP1B/LRP1. On the contrary, the clade composed of LRX + 1/LRP5/LRP6 did not show significant bootstrap value indicating that these relationships were not reliable. However, this clade grouped with LRP2/LRP1B/LRP1 and LRP4 and all together shared a common ancestor with VLDLR/LDLR/LRP8. None of the identified sequences belonging to *C. orientalis* was located in the LRP1B and LRP4 clades. Since the gene sequences encoding these two proteins were found in *P. waltl* genome, the other salamander considered, the lack of identification in *C. orientalis* might be due to the absence of transcriptional activity for these two genes in the transcriptomes here analyzed.

### Protein structure prediction and comparison

Based on the phylogenetic analysis results, we made the decision to determine the structure of basal sarcopterygian VTGR (*L. menadoensis*, *P. annectens*, and *P. waltl*) and those of LDLR and LRP8 sequences of *C. orientalis.* Models were used to carry out multiple sequence alignments to evaluate the conservation level of secondary structures (Supplementary File S2). Our findings showed that the VTGR in the four basal sarcopterygians was conserved and eight β-hairpin motifs were found at the N-terminal (Supplementary File S2). This structural region appeared to be also conserved in LDLR and LRP8 receptors of *C. orientalis.* However, in the case of LDLR, the lack of the first β-sheet, which constitutes the first β-hairpin structure, was highlighted (Supplementary File S3).Moreover, due to the higher similarity between LRP8 and VLDLR, the protein structural organization of *C. orientalis* LRP8 was compared with orthologs of species considered in phylogenetic analysis highlighting a conservation in terms of protein domain organization (Supplementary File S4).

### Gene expression analyses

**Fig. 3:**
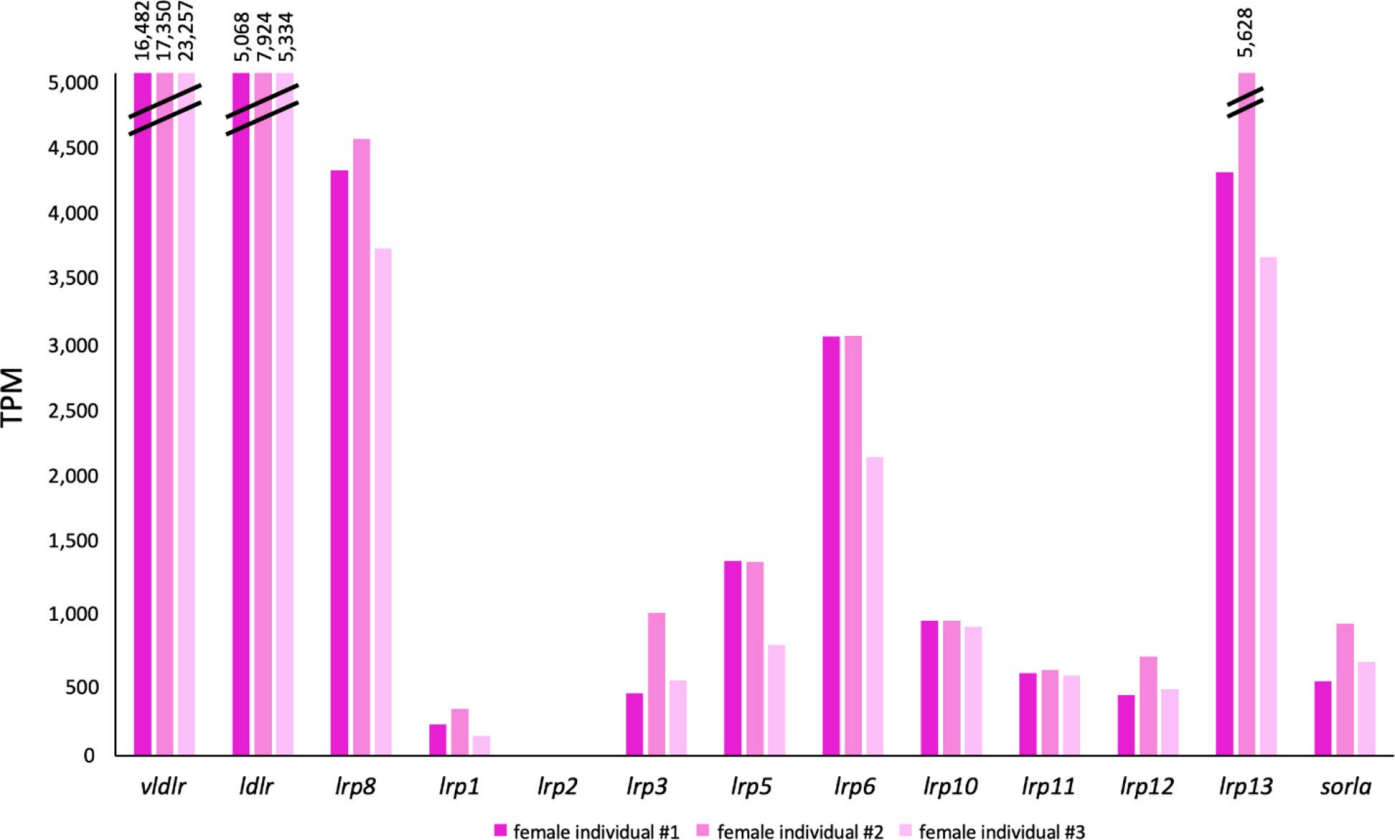
Transcriptional activity of members belonging to the LDLR superfamily in the three replicates of *Cynops orientalis* female gonad. Expression values of the low density lipoprotein receptor (LDLR) superfamily members obtained for the three replicates of *Cynops orientalis* female gonad. Values are reported as transcripts per million kilobases (TPM).

**Fig. 4:**
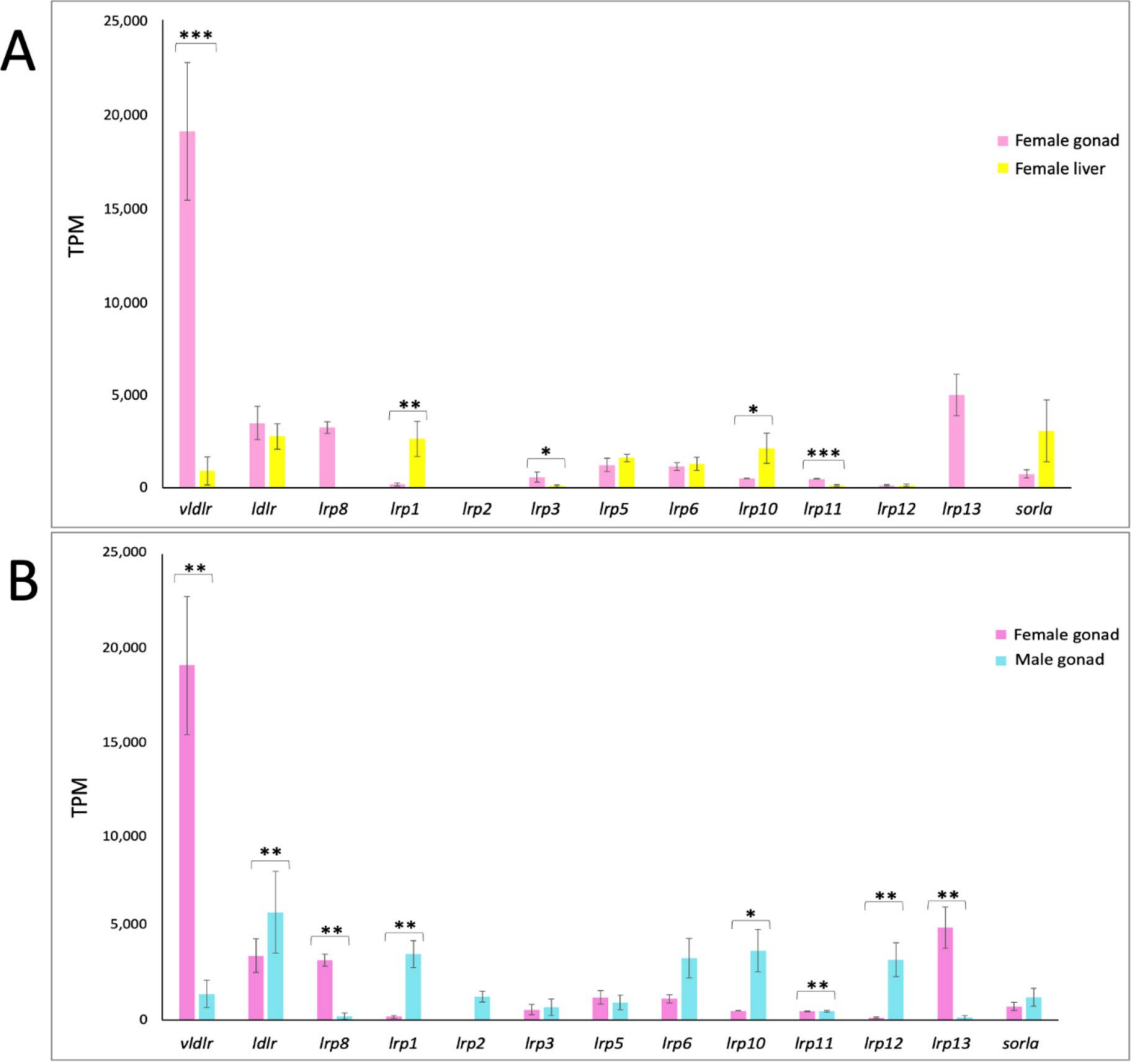
Transcriptional activity of members belonging to the low density lipoprotein receptor (LDLR) superfamily in *Cynops orientalis* between gonad and liver of female specimens (**A**) and between female and male gonads (**B**). (**A**) Expression values of the 13 members of the Low Density Lipoprotein Receptor (LDLR) superfamily in *Cynops orientalis*. Means and standard deviations were obtained from the triplicates of the female gonad (pink) and female liver (yellow) *Cynops orientalis* transcriptomes. Values are reported as transcripts per million kilobases (TPM). (**B**) Expression values of the 13 members of the Low Density Lipoprotein Receptor (LDLR) superfamily in *Cynops orientalis*. Means and standard deviations were obtained from the triplicates of the female (pink) and male gonad (light blue) *Cynops orientalis* transcriptomes. Values are reported as transcripts per million kilobases (TPM). Statistically significant differences are presented as *for *p* > 0.05, ** for *p* < 0.01, and *** for *p* < 0.001.

The expression analysis of the 15 genes belonging to the low-density protein receptors superfamily was conducted in the transcriptomes of *C. orientalis* obtained from female liver and gonadal tissues of both sexes. Overall, in the female gonad the higher transcriptional level was reported for *vldlr*,* lrp8*,* lrp13*, and *ldlr* (Fig. 3). Considering the comparison between female gonad and liver, the highest transcriptional activity was reported for *vldlr* in the gonad, with statistically significant values. It was noteworthy the absence of *lrp8* and *lrp13* in the hepatic tissue (Fig. 4A). Moreover, regarding the gene expression profiles obtained in female and male gonads, statistically significant higher values were obtained for *vldlr*, *lrp8*, and *lrp13* in ovary (Fig. 4B) while for *ldlr* in testis. Concerning the expression values of *vldlr* gene in the three female gonad replicates, the highest transcriptional activity was recorded in the third female individual (Fig. 3).

## Discussion

The Vtg receptor, belonging to the LDLR superfamily^21,39^, is fundamental for the vitellogenin endocytosis within the oocytes^40^. In particular, the receptors belonging to this superfamily are cell surface proteins, important for the transport of various ligands across cell membranes. Most of these are not only involved in endocytosis, but also in the transduction of important signals during embryonic development and in the regulation of cholesterol homeostasis^27^. In tetrapods, information on the LDLR superfamily and Vtg receptors are limited to a few taxa^35^. Therefore, gene characterization studies of these receptors in the newt *C. orientalis* and in other basal sarcopterygians might deepen our knowledge on the evolution of these proteins and their roles. Our analysis allowed us to identify 13 complete sequences in *C. orientalis*, eight sequences in *L. menadoensis*, and nine sequences in *P. annectens*. The phylogenetic analysis confirmed the orthology of the candidate sequences to the Vtg and LDLR superfamily receptors. Moreover, the position of these sequences in the phylogenetic tree helped us to unravel the evolutionary history of these genes. Given the presence of all 15 genes belonging to the LDLR superfamily both in actinopterygians and sarcopterygians, this gene set was already present in their common ancestor originated in the Ordovician era (510 mya) and that already experienced two rounds of WGD (1R and 2R^)41^. Furthermore, duplicate genes were detected in actinopterygians (*lrp1*, *lrp13*, *lrp8*, and *lrp2*). In particular, microsyntenic analyses revealed that the two copies of *lrp1*, *lrp8*, and *lrp13* in *T. rubripes* were located on separate chromosomes known to be originated by teleost specific WGD 3R estimated to have occurred between 226 and 350 mya^41^. Differently, the duplication event of *lrp2* cannot be attributable to the 3R WGD event, given the presence of two copies also in the non-teleost *L. oculatus*. Therefore, these two copies were already present in the common ancestor of spotted gar and pufferfish, in the Devonian era (409 mya)^41^.

Interestingly, the domain organization of the receptors considered in the present study corroborated results obtained through phylogenetic analysis (Fig. 1). Indeed, LRP1, 1B, and 2, which grouped together, showed a very similar protein domain organization; the same result was also observed for the groups of receptors LRP3/LRP10/LRP12 and VLDLR/LDLR/LRP8. The monophyletic relationship observed for these two clades has also been highlighted in previous phylogenetic analyses^23,32,34^. Therefore, the common origin of the considered sequences allowed us to exclude structural similarity due to convergent evolution. Moreover, VLDLR, LDLR, and LRP8 structural models showed a conserved alignment of secondary structure elements in *C. orientalis*. This suggested that the function of the three proteins could be similar.

LDLR has been reported to be involved in the lipid transfer within maturing oocytes in the salmonid *Oncorhynchus clarki*^33^. However, the lack of the first beta sheet in the N-terminus secondary structure element belonging to LBR and known to be fundamental for the interaction between vitellogenin receptor and its ligand^30,31^, suggested that it might not function as vitellogenin receptor. Moreover, the transcriptional activity of the *ldlr* gene showed comparable levels in the two female tissues and was higher in testis than in ovary in *C. orientalis*. This latter evidence agreed with the recently published papers in which LDLR has been associated with the regulation of lipid homeostasis in normal male reproductive function^42,43^.

At the gonadal level, LRP8 is involved in the formation of cholesterol storage, also useful for the synthesis of steroids during the folliculogenesis^44^ and in the achieving of the right weight of the yolk and albumen^45^. Recently, Akhavan and colleagues (2020)^46^ have proposed that LRP8 might mediate ovarian yolk and lipid uptake in the beluga sturgeon *Huso huso L*. Therefore, a possible interaction of LRP8 with Vtg or other lipoprotein particles might be hypothesized also in *C. orientalis*.

Indeed, analyzing transcriptomic data, *lrp8* as *vldlr* and *lrp13*, involved in vitellogenesis, showed a greater expression in the female gonad compared to the female liver and the male gonad in the newt. Moreover, the high degree of conservation observed aligning secondary structures of LRP8 between considered species suggested a similar function in oviparous and ovoviviparous species. The different transcriptional activity reported for the three genes *vldlr*, *lrp8*, and *lrp13* might be related to the variable expression levels of the diverse forms of *vtgs*, evidenced by Carducci and collaborators (2021)^13^ for the same specimens here analyzed. Moreover, the different transcriptional activity of the *vldlr* gene in the three female individuals can be explained considering gonadal histological observations^47^ and *vtg* expression levels^13^. Indeed, gonads of the three females were characterized by a clear asynchronous maturation stage^47^. Of these, the third one was reported as less mature than others, in agreement with the lower transcriptional level reported for the *vtg* and the highest value obtained for the *vtg* receptor. This scenario suggested that the gonad is producing receptor to receive Vtg, whose production has just begun in the liver. In *O. mykiss*, the expression of *vtgr* mRNA starts in the pre-vitellogenic stage of oocytes and diminishes at the beginning of the vitellogenesis^48^. On the contrary, the highest transcriptional value detected for the *vtg* and the lower value for the *vtg* receptor were obtained for the first *C. orientalis* female specimen, having mature gonads.

## Conclusion

In this work, we report the characterization of the VTGR in the newt *C. orientalis* and an extensive phylogenetic analysis of the LDLR superfamily receptors that helped unravel the evolutionary history of these genes. Our results revealed that the gene set of this superfamily was present in the common ancestor of sarcopterygians and actinopterygians and remained conserved throughout these evolutionary lineages. Moreover, combining phylogenetic analysis, secondary structure predictions, and gene expression analyses, our findings supported the role of VTGR as vitellogenin oocyte membrane receptor in the newt. The close relationship of LRP8 with VLDLR in the tree and expression of these genes in *C. orientalis* ovary allowed us to propose that also LRP8 could be involved in vitellogenesis.

## Materials and methods

### *Vitellogenin* receptor sequences identification and characterization

In this study, 11 genera belonging to 10 orders were considered: Coelacanthiformes (*L. menadoensis* and *L. chalumnae*), Ceratodontiformes (*P. annectens*), Lepisosteiformes (*Lepisosteus oculatus*), Tetraodontiformes (*Takifugu rubripes*), Caudata (*C. orientalis* and *P. waltl*), Anura (*Xenopus tropicalis*), Testudines (*Pelodiscus sinensis*), Galliformes (*Gallus gallus*), Rodentia (*Mus musculus*), and Primates (*Homo sapiens*).

Analyses on *L. menadoensis*, *P. annectens*, and *C. orientalis* were carried out using transcriptomes obtained by the Molecular Phylogenetic laboratory of the Polytechnic University of Marche^47,49,50^ (accession numbers: PRJNA175365, PRJNA164839, PRJNA574599, respectively); while, for the remaining organisms, the search was performed using the transcriptomes available in NCBI (https://www.ncbi.nlm.nih.gov) and the genomic information present in ENSEMBL (https://www.ensembl.org/index.html). In detail, for *C. orientalis* a search using BLAST + v2.11.0^51^ was performed, setting protein sequences of the evolutionary related species *X. tropicalis* as queries. Nucleotide sequences corresponding to subjects with the highest score were then translated and the correctness checked through a BLASTP search. The same procedure was used for *L. menadoensis* and *P. annectens*. The coding sequence of each transcript identified as potential candidate of this gene superfamily was then characterized using Expasy translate tool (SIB, www.expasy.org). To complete the sequences of *L. menadoensis* not retrieved at the transcriptome-level, the genome of *L. chalumnae*, available in NCBI, was used (GCF_000225785.1). Moreover, the analysis of this superfamily was extended to other organisms: *L. chalumnae*, *L. oculatus*, *T. rubripes*, *P. waltl*, *X. tropicalis*, *P. sinensis*, *G. gallus*, *M. musculus*, and *H. sapiens* (Supplementary Table S1). Sequences obtained in this study were deposited at GenBank under the accession numbers PP516878:PP516907. Selected sequences were then used to generate preliminary phylogenetic trees, to confirm their sequence orthology.

### Phylogenetic analysis

In order to identify and evaluate evolutionary distances of VTGRs of organisms considered in this study, a phylogenetic analysis was conducted. Sequences obtained from the transcriptomes of *L. menadoensis*, *P. annectens*, and *C. orientalis* were aligned with another 131 sequences of the other organisms included in the present study, retrieved in NCBI and ENSEMBL public repositories (the complete list of accession numbers of sequences used was reported in Supplementary Table S1). The alignment was performed with ClustalW^52^ with default parameters. The phylogenetic analysis was carried out with RAxML-NG^53^ with 1000 bootstraps. ITOL was used for tree visualization^54^. The JTT + G4 model was defined as the most appropriate model using ModelTest-NG^55^.

### Microsyntenic analysis in actinopterygians and basal sarcopterygians

The microsyntenic arrangement of *lrp1*, *lrp2*, *lrp8*, and *lrp13* genes was obtained combining information retrieved from NCBI Gene, Genomicus, and Ensembl to determine upstream and downstream flanking genes of those of interest.

### Protein structure prediction and comparison

The three-dimensional (3D) model prediction for VTGR, LDLR, and LRP8, was built using Swiss-Model^56^ web server, available online (http://swissmodel.expasy.org). Global Model Quality Estimate (GMQE) and Quality Model Energy ANalysis (QMEAN) parameters were used to have an estimate of the quality of the model globally and per residue. The 3D predictions were used to determine the secondary structures present along the aminoacidic sequences of the proteins analyzed. The secondary protein structures obtained for VTGR, LDLR, and LRP8 were compared using 2DSS server^57^.

### Gene expression analyses

*C. orientalis* trimmed reads of RNA-seq data of the three female livers and the three male and female gonads were mapped against *ldlr* superfamily genes in CLC Genomics Workbench v.12 environment (Qiagen, Hilden, Germany) using the *RNA-seq* mapping tool, setting parameters as highly stringent (length fraction 0.75 and similarity fraction 0.8). Gene expression levels were computed as Transcripts Per Million (TPM), as this metric allows to efficiently compare gene expression levels both within and between samples. We followed the same strategy described in Biscotti et al. (2016)^50^. In summary, trimmed sequencing reads were aligned to the transcriptome using RNA-seq mapping tools in CLC Genomics Workbench v.24.0.1 (Qiagen, Hilden, Germany). The mapping parameters were set at length fraction and a similarity fraction of 0.80. Gene expression levels, reported as transcripts per million (TPM), were calculated using a scaling factor derived from the cumulative expression of 3,354 unequivocal single-copy orthologs shared between analyzed tissues. Statistically significant differences were determined using one-way ANOVA, with significance denoted by *for *p* > 0.05, ** for *p* < 0.01, and *** for *p* < 0.001.

## Electronic supplementary material

Below is the link to the electronic supplementary material.

Supplementary Material 1

Supplementary Material 2

## Data Availability

The datasets generated during the current study are available in the Genbank repository under the accessions PP516878:PP516907. Details about all data analysed during the current study are provided in Supplementary Table S1.
