# Supplementary material for: A bioinformatic approach to characterize the vitellogenin receptor and the low density lipoprotein receptor superfamily in the newt Cynops orientalis
Source: Sci Rep. 2025 Jan 27;15:3403. doi: 10.1038/s41598-025-88011-6 (PMC11772764; doi:10.1038/s41598-025-88011-6)
Supplement: Supplementary file 1 — Supplementary Material 1 [file 41598_2025_88011_MOESM1_ESM.pdf]

Supporting Information

A bioinformatic approach to characterize the vitellogenin receptor and the Low Density Lipoprotein Receptor superfamily in the newt *Cynops orientalis*

Chiara Spinsante†, Federica Carducci†, Elisa Carotti, Adriana Canapa, Davide Bizzaro, Maria Assunta Biscotti*, and Marco Barucca

Department of Life and Environmental Sciences, Polytechnic University of Marche, Via Brecce Bianche 60131, Ancona (Italy)

†These authors contributed equally to this work.

*Corresponding author: e-mail: m.a.biscotti@univpm.it

Supplementary File S1: **Microsyntenic analyses. (A)** Microsyntenic arrangement of *lrp1* gene in basal sarcopterygians (lungfish and coelacanth) and in actinopterygians (pufferfish and spotted gar). Triangles indicate genes and their direction. Colored triangles are the only shared genes between species considered in this analysis. Black-filled triangles indicate *lrp1* gene. Note that gene distances are not in scale. The dashed line triangles indicate uncharacterized genes. **(B)** Microsyntenic arrangement of *lrp2* gene in basal sarcopterygians (lungfish and coelacanth) and in actinopterygians (pufferfish and spotted gar). Triangles indicate genes and their direction. Colored triangles are the only shared genes between species considered in this analysis. Black-filled triangles indicate *lrp2* gene. Note that gene distances are not in scale. The dashed line triangles indicate uncharacterized genes. For these latter, if possible, protein name was indicated below the LOC identification number. **(C)** Microsyntenic arrangement of *lrp8* gene in basal sarcopterygians (lungfish and coelacanth) and in actinopterygians (pufferfish and spotted gar). Triangles indicate genes and their direction. Colored triangles are the only shared genes between species considered in this analysis. Black-filled triangles indicate *lrp8* gene. Note that gene distances are not in scale. The dashed line triangles indicate uncharacterized genes. **(D)** Microsyntenic arrangement of *lrp13* gene in basal sarcopterygians (lungfish and coelacanth) and in actinopterygians (pufferfish and spotted gar). Triangles indicate genes and their direction. Colored triangles are the only shared genes between species considered in this analysis. Black-filled triangles indicate *lrp13* gene. Note that gene distances are not in scale. The dashed line triangles indicate uncharacterized genes. The following manual reconstruction was made combining information from NCBI, ENSEMBL, and Genomicus databases.

Supplementary File S2. Basal sarcopterygians VTGR secondary structure alignments comparisons. Secondary structure alignments comparisons of *Cynops orientalis* vitellogenin receptor (VTGR) with those of other basal sarcopterygians *Pleurodeles waltl*, *Protopterus annectens*, and *Latimeria menadoensis.* Colored characters refer to the primary amino acid sequence, red lines indicate the non-structured traits of the amino acid sequence, the black-colored waiving lines represent alpha helices, yellow-colored arrows indicate beta sheets.

Supplementary File S3. VTGR, LRP8, and LDLR secondary structure alignments comparisons. Secondary structure alignments comparisons of *Cynops orientalis* vitellogenin receptor (VTGR), LDL receptor-related protein 8 (LRP8), and Low Density Lipoprotein Receptor (LDLR). Colored characters refer to the primary amino acid sequence, red lines indicate the non-structured traits of the amino acid sequence, the black-colored waiving lines represent alpha helices, yellow-colored arrows indicate beta sheets.

Supplementary File S4. Secondary structure alignments comparisons between *Cynops orientalis* LDL receptor-related protein 8 (LRP8) with the other organisms considered in the present study. In particular, comparisons were made for *C. orientalis* (Cori) vs *Latimeria chalumnae* (Lcha), *C. orientalis* (Cori) vs *Protopterus annectens* (Pann), *C. orientalis* (Cori) vs *Lepisosteus oculatus* (Locu), *C. orientalis* (Cori) vs *Takifugu rubripes* (Trub, for both duplicates), *C. orientalis* (Cori) vs *Pleurodeles waltl* (Pwal), *C. orientalis* (Cori) vs *Xenopus tropicalis* (Xtro), *C. orientalis* (Cori) vs *Pelodiscus sinensis* (Psin), *C. orientalis* (Cori) vs *Gallus gallus* (Ggal), *C. orientalis* (Cori) vs *Mus musculus* (Mmu), and *C. orientalis* (Cori) vs *Homo sapiens* (Hsap). Characters refer to the primary amino acid sequence, red lines indicate the non-structured traits of the amino acid sequence, the black-colored waiving lines represent alpha helices, yellow-colored arrows indicate beta sheets.

Supplementary Table S1. Details of gene sequences identified and accession numbers of raw data. For each organism considered in the present study, details of gene sequences identified, together with accession numbers of raw data downloaded from public repositories are provided.
