# Supplementary material for: A bioinformatic approach to characterize the vitellogenin receptor and the low density lipoprotein receptor superfamily in the newt Cynops orientalis
Source: Sci Rep. 2025 Jan 27;15:3403. doi: 10.1038/s41598-025-88011-6 (PMC11772764; doi:10.1038/s41598-025-88011-6)
Supplement: Supplementary file 2 — Supplementary Material 2 [file 41598_2025_88011_MOESM2_ESM.zip › Supporting Information/FileS1.pdf]

Supplementary File S1: Microsyntenic analyses.

(A)

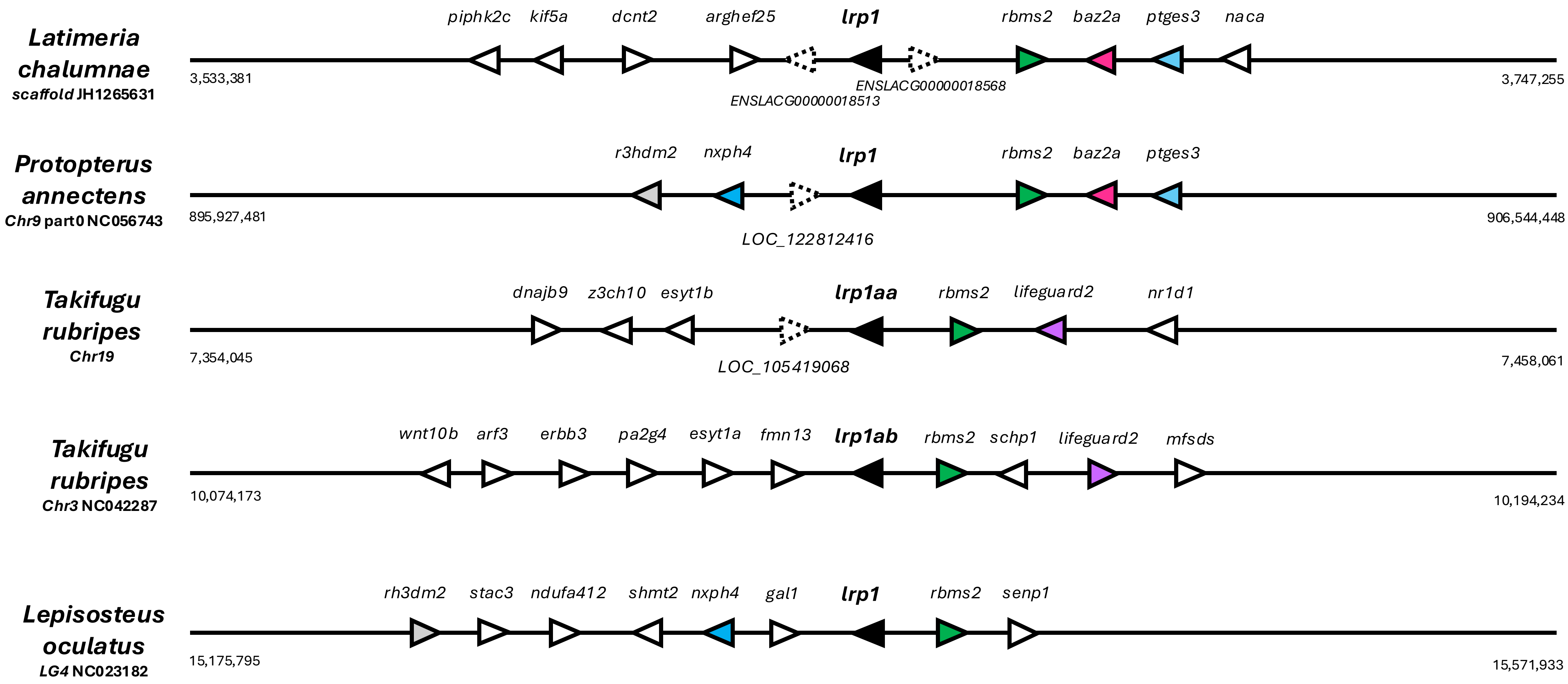

(A) Microsyntenic arrangement of lrp1 gene in basal sarcopterygians (lungfish and coelacanth) and in actinopterygians (pufferfish and spotted gar). Triangles indicate genes and their direction. Colored triangles are the only shared genes between species considered in this analysis. Black - filled triangles indicate lrp1 gene. Note that gene distances are not in scale. The dashed line triangles indicate uncharacterized genes.

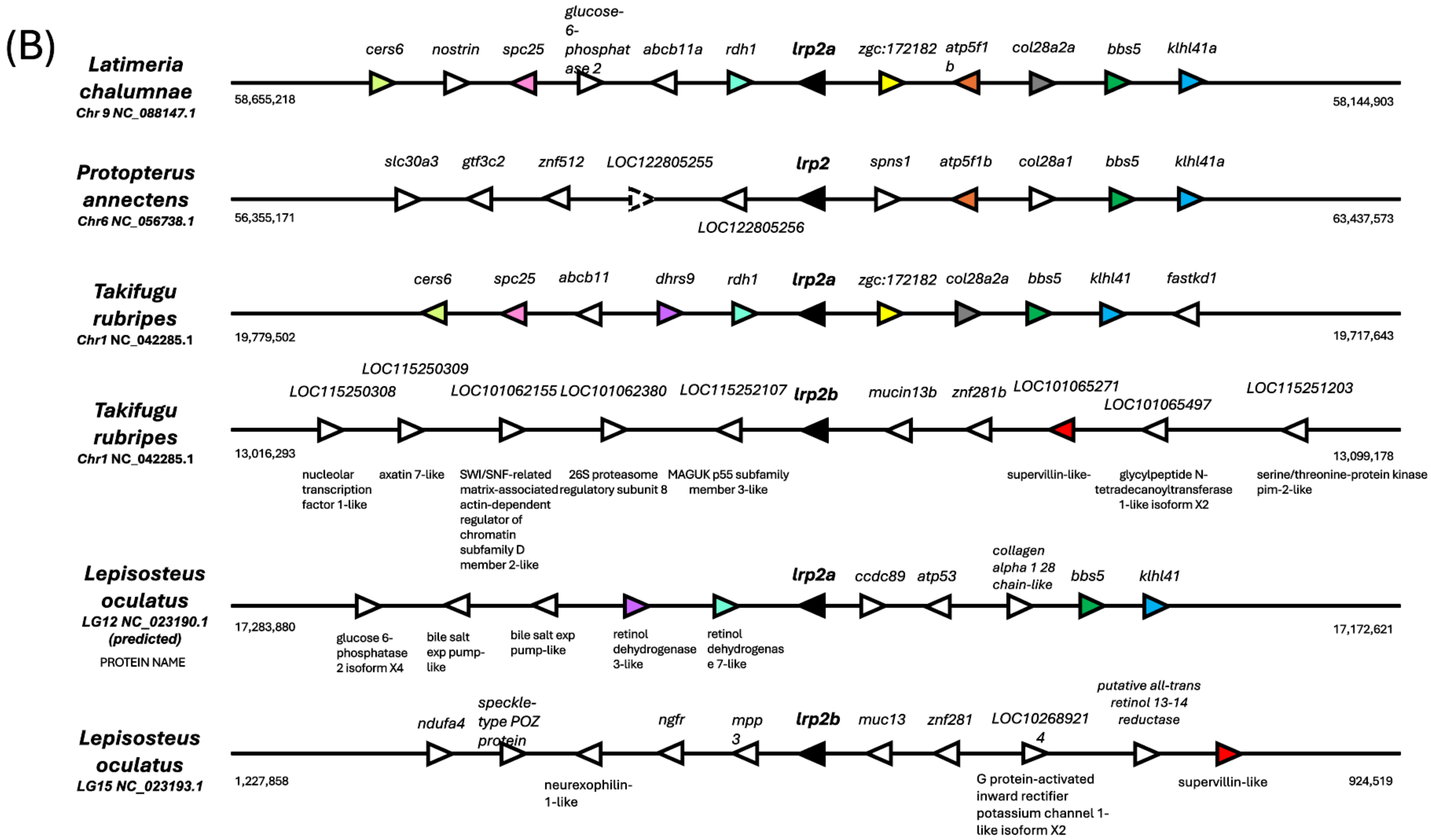

(B) Microsyntenic arrangement of lrp2 gene in basal sarcopterygians (lungfish and coelacanth) and in actinopterygians (pufferfish and spotted gar). Triangles indicate genes and their direction. Colored triangles are the only shared genes between species considered in this analysis. Black - filled triangles indicate lrp2 gene. Note that gene distances are not in scale. The dashed line triangles indicate uncharacterized genes. For these latter, if possible, protein name was indicated below the LOC identification number.

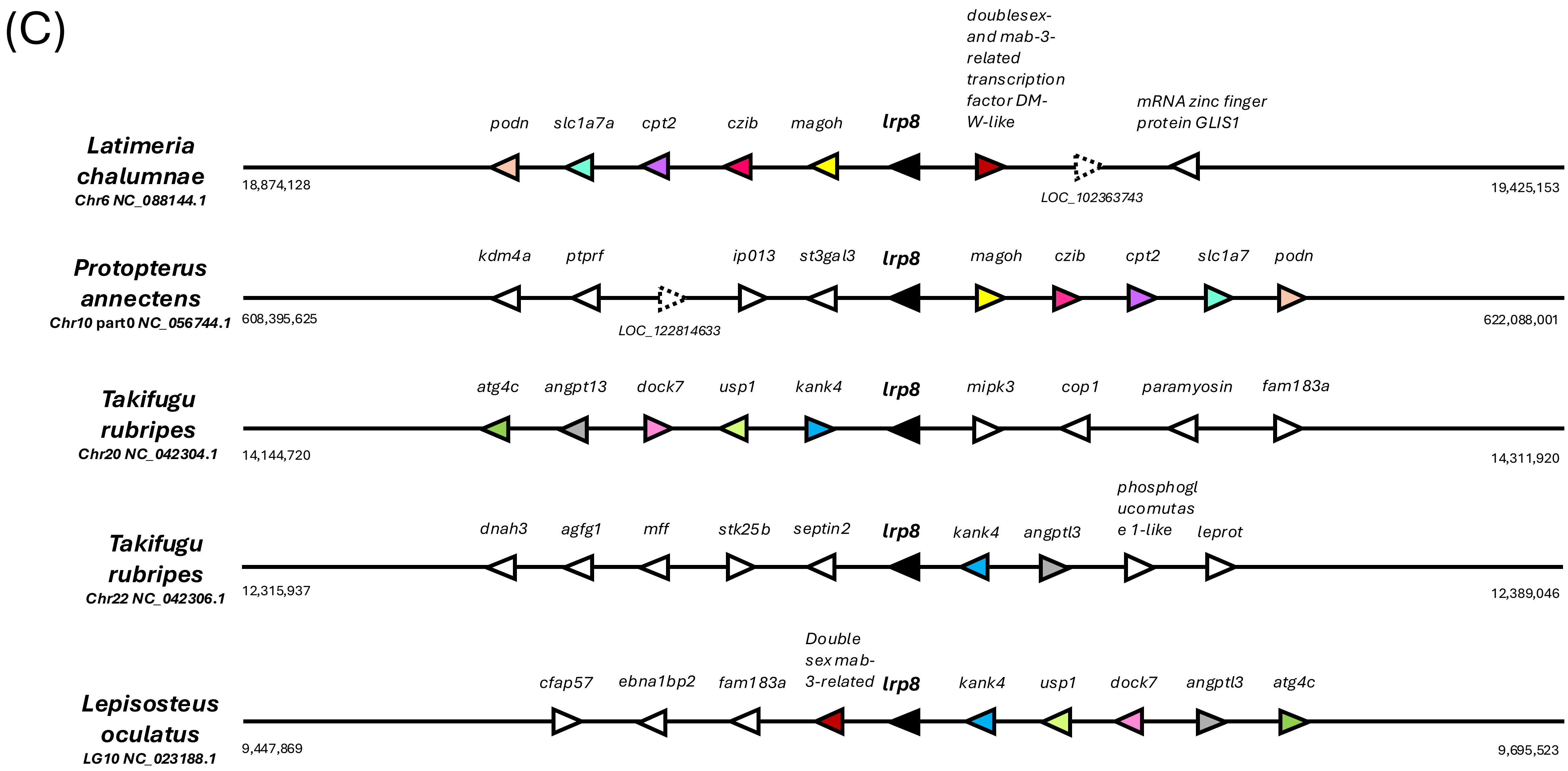

(C) Microsyntenic arrangement of lrp8 gene in basal sarcopterygians (lungfish and coelacanth) and in actinopterygians (pufferfish and spotted gar). Triangles indicate genes and their direction. Colored triangles are the only shared genes between species considered in this analysis. Black - filled triangles indicate lrp8 gene. Note that gene distances are not in scale. The dashed line triangles indicate uncharacterized genes.

(D)

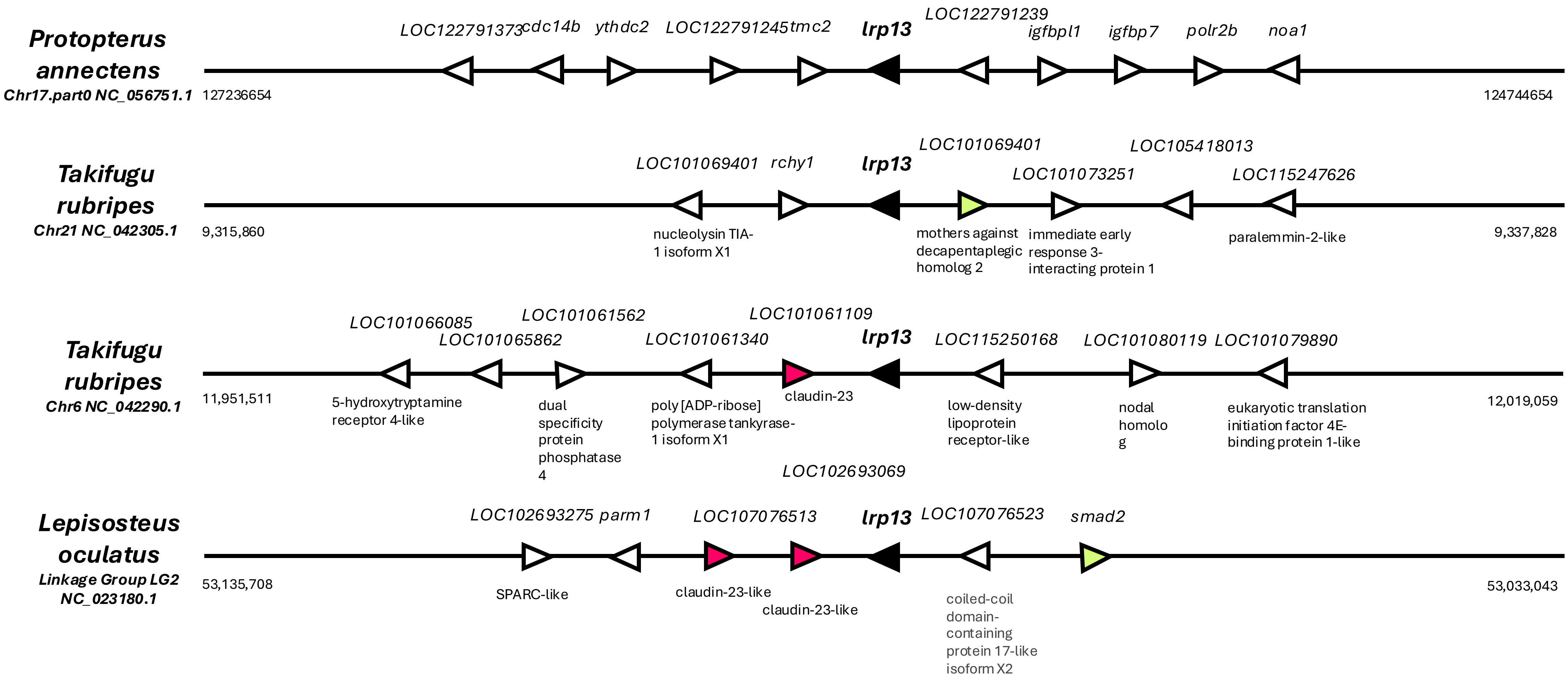

(D) Microsyntenic arrangement of lrp13 gene in basal sarcopterygians (lungfish and coelacanth) and in actinopterygians (pufferfish and spotted gar). Triangles indicate genes and their direction. Colored triangles are the only shared genes between species considered in this analysis. Black - filled triangles indicate lrp13 gene. Note that gene distances are not in scale. The dashed line triangles indicate uncharacterized genes. The following manual reconstruction was made combining information from NCBI, ENSEMBL, and Genomicus databases.
