# Supplementary material for: A bioinformatic approach to characterize the vitellogenin receptor and the low density lipoprotein receptor superfamily in the newt Cynops orientalis
Source: Sci Rep. 2025 Jan 27;15:3403. doi: 10.1038/s41598-025-88011-6 (PMC11772764; doi:10.1038/s41598-025-88011-6)
Supplement: Supplementary file 2 — Supplementary Material 2 [file 41598_2025_88011_MOESM2_ESM.zip › Supporting Information/FileS3.pdf]

2dSS secondary structure visualization

View 2d Alignment

Supplementary File S3. VTGR, LRP8, and LDLR secondary structure alignments comparisons. Secondary structure alignments comparisons of Cynops orientalis vitellogenin receptor (VTGR), LDL receptor-related protein 8 (LRP8), and Low Density Lipoprotein Receptor (LDLR).

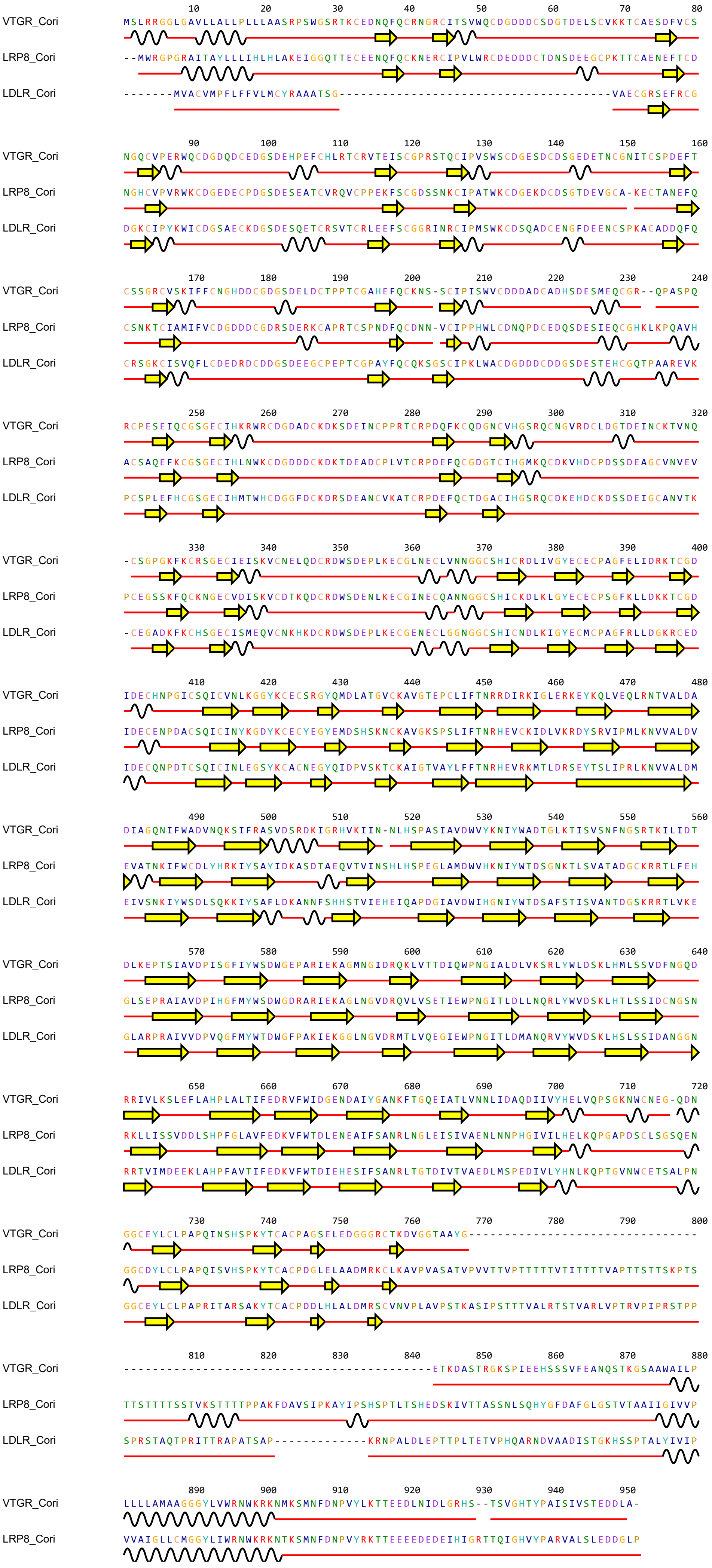

LEGEND
- characters: primary amino acid sequence
- red lines: non-structured traits of the amino acid sequence
- black-colored waiving lines: alpha helices
- yellow-colored arrows: beta sheets

LDLR_Cori LAVLCLVAFSAFLLWKKWRLKNTNSINFDNPVYQKTTEDEIHICRNQDG------YTYPSRQMVSVEDDVA-

download svg file : here

previous

External Links

Cite 2dSS

If you use 2dSS, please cite:.

Contact Us

For questions, comments, or suggestions feel free to contact us.

Click here

Copyright © LCQB. All Rights Reserved

Design by J. Bernardes inspired in DreamTemplate
