# Supplementary material for: A bioinformatic approach to characterize the vitellogenin receptor and the low density lipoprotein receptor superfamily in the newt Cynops orientalis
Source: Sci Rep. 2025 Jan 27;15:3403. doi: 10.1038/s41598-025-88011-6 (PMC11772764; doi:10.1038/s41598-025-88011-6)
Supplement: Supplementary file 2 — Supplementary Material 2 [file 41598_2025_88011_MOESM2_ESM.zip › Supporting Information/FileS2.pdf]

2dSS secondary structure visualization

View 2d Alignment

Supplementary File S2. Basal sarcopterygians VTGR secondary structure alignments comparisons. Secondary structure alignments comparisons of Cynops orientalis vitellogenin receptor (VTGR) with those of other basal sarcopterygians Pleurodeles waltl, Protopterus annectens, and Latimeria menadoensis.

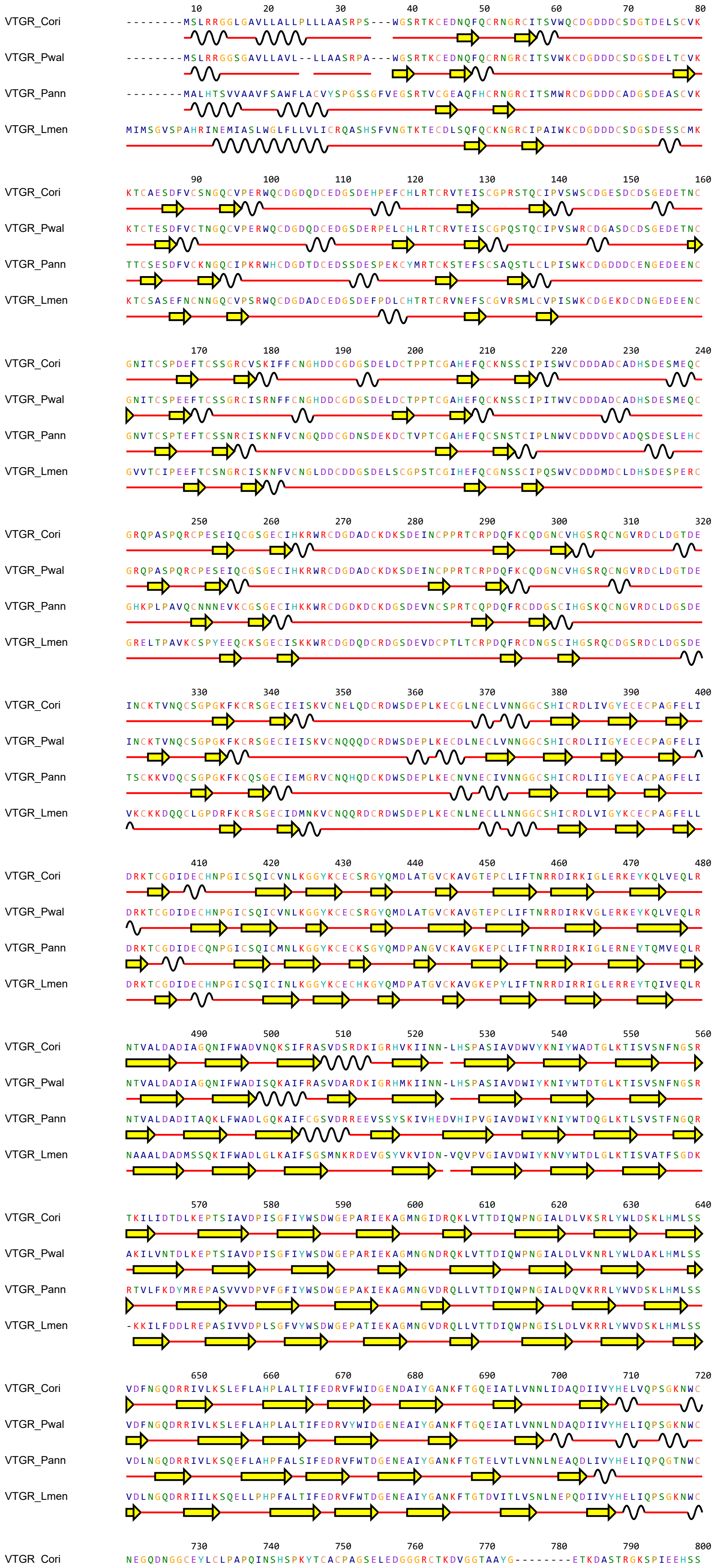

LEGEND
- characters: primary amino acid sequence
- red lines: non-structured traits of the amino acid sequence
- black-colored waiving lines: alpha helices
- yellow-colored arrows: beta sheets

```
VTGR_Pwal    SEGQDNGGCEYLCLPAPQINSYSPKYTCACPAGSELENGGGRCTKEVGGTADYG--------ETRDASTRGKSQIEEHSS

VTGR_Pann    KEELENGGCEYLCLPAPQINSRSPKYTCACPVGKELHNDGRRCRRAAASAKIPLGHEGNDQRLLMNLPTAGSVTGESNMS

VTGR_Lmen    NEHSKNGGCEYLCLPAPQINNRSPKFTCACPTGIELEKDGQKCKATVPVTGISEVKT-KEYTTLSWLPTVGPHPGEQNLS


                       810       820       830       840       850       860       870       880
VTGR_Cori    SVFEANQSTKG-SAAWAILPLLLLAMAAGGGYLVWRNWKRKNMKSMNFDNPVYLKTTEEDLNIDLGRHSTSVGHTYPAIS

VTGR_Pwal    SVFEANQSTKGSSAAWAILPLLLLAMAAAGGYLVWRNWKRKNMKSMNFDNPVYLKTTEEDLNIDLGRHSSSVGHTYPAIS

VTGR_Pann    SIYDANTSTRSSSAAWAILPIMLIAVSALGGYIVWRNWQRKNMKSMNFDNPVYLKTTDDDLTVDISRHSGAVGHTYPAIS

VTGR_Lmen    SVHEVN-AAKGLTGAWAVLPIFLLAVGAAAGYLVWRNWQQKNMKSMNFDNPVYLKTTEEDLAIDISRHASSVGHTYPAIS


VTGR_Cori    IVSTEDDLA

VTGR_Pwal    IVSTEDDLA

VTGR_Pann    VVNTEDDLA

VTGR_Lmen    VVNTEDDQA
```

download svg file : here

previous

External Links

Cite 2dSS

If you use 2dSS, please cite:.

Contact Us

For questions, comments, or suggestions feel free to contact us.

Click here

Copyright © LCQB. All Rights Reserved

Design by J. Bernardes inspired in DreamTemplate
