# Supplementary material for: A bioinformatic approach to characterize the vitellogenin receptor and the low density lipoprotein receptor superfamily in the newt Cynops orientalis
Source: Sci Rep. 2025 Jan 27;15:3403. doi: 10.1038/s41598-025-88011-6 (PMC11772764; doi:10.1038/s41598-025-88011-6)
Supplement: Supplementary file 2 — Supplementary Material 2 [file 41598_2025_88011_MOESM2_ESM.zip › Supporting Information/FileS4.pdf]

Supplementary File S4. Secondary structure alignments comparisons between Cynops orientalis LDL receptor-related protein 8 (LRP8) with the other organisms considered in the present study.

2dSS secondary structure visualization

Comparison between C. orientalis (Cori) and Latimeria chalumnae (Lcha)

View 2d Alignment

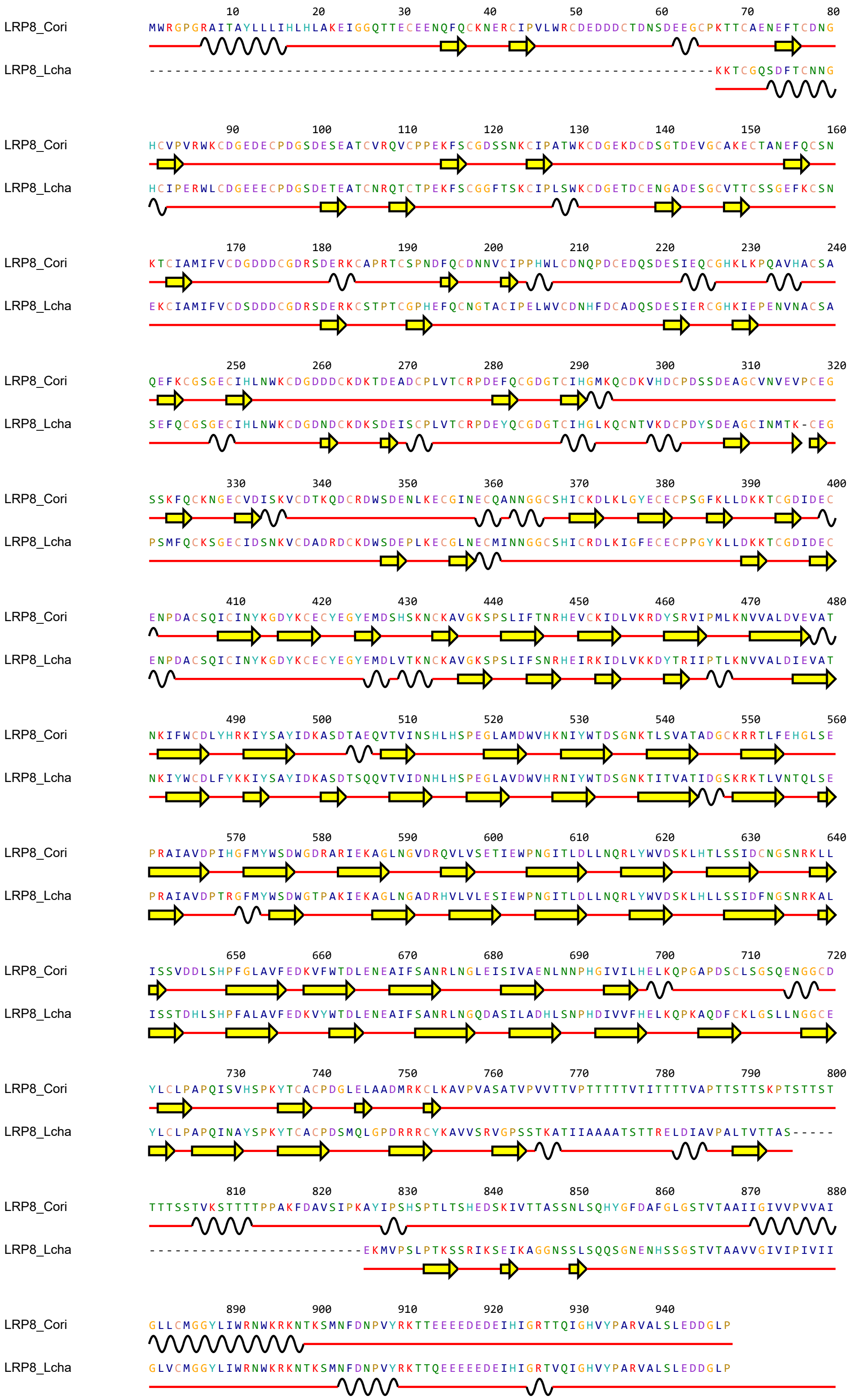

LEGEND
- characters: primary amino acid sequence
- red lines: non-structured traits of the amino acid sequence
- black-colored waiving lines: alpha helices
- yellow-colored arrows: beta sheets

download svg file : here

previous

External Links

Cite 2dSS

If you use 2dSS, please cite:.

Contact Us

For questions, comments, or suggestions feel free to contact us.

Click here

Copyright © LCQB. All Rights Reserved

Design by J. Bernardes inspired in DreamTemplate

2dSS secondary structure visualization

Comparison between C. orientalis (Cori) and Protopterus annectens (Pann)

View 2d Alignment

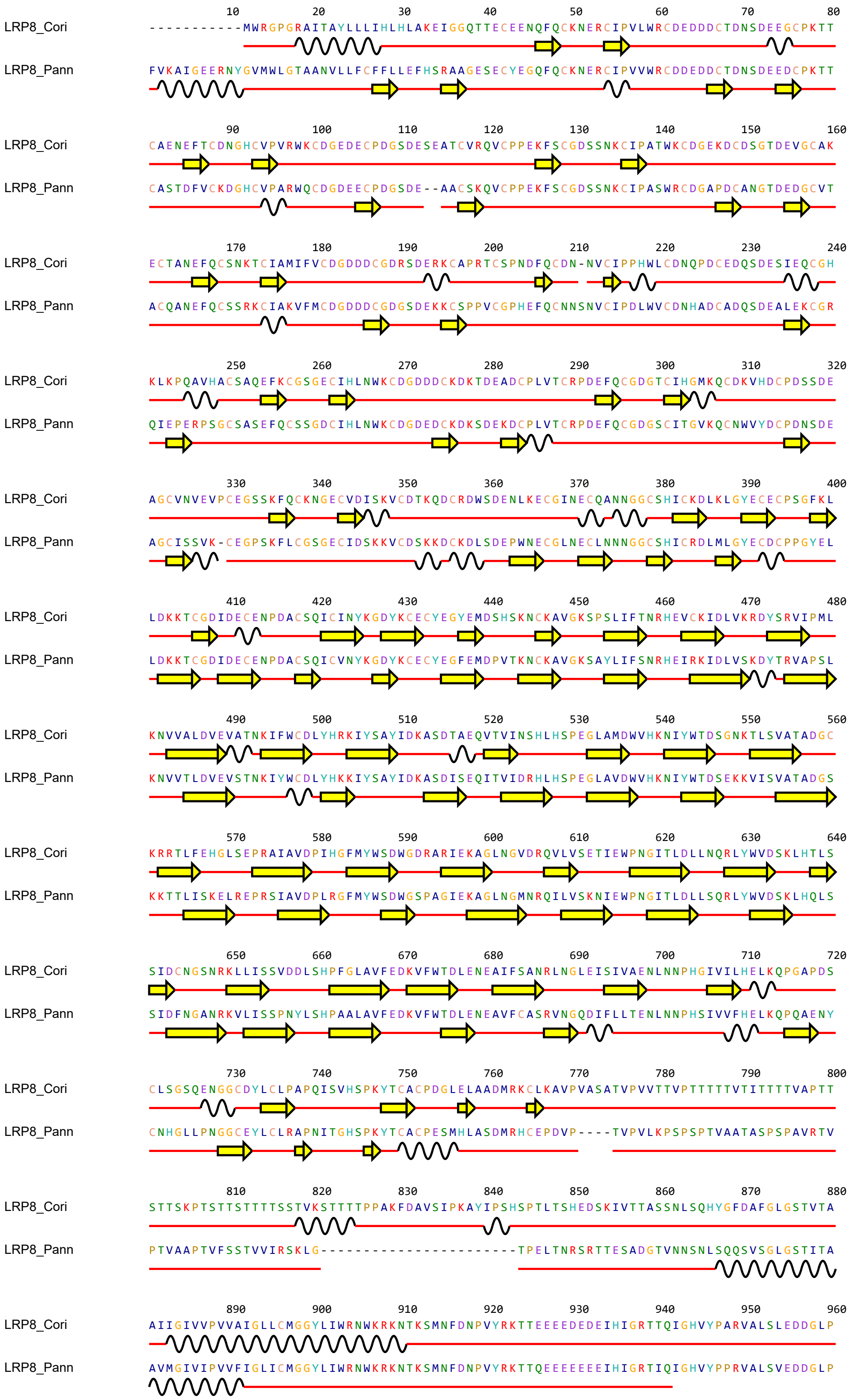

LEGEND
- characters: primary amino acid sequence
- red lines: non-structured traits of the amino acid sequence
- black-colored waiving lines: alpha helices
- yellow-colored arrows: beta sheets

download svg file : here

previous

External Links

Cite 2dSS

If you use 2dSS, please cite:.

Contact Us

For questions, comments, or suggestions feel free to contact us.

Click here

Copyright © LCQB. All Rights Reserved

Design by J. Bernardes inspired in DreamTemplate

2dSS secondary structure visualization

View 2d Alignment

Comparison between C. orientalis (Cori) and Lepisosteus oculatus (Locu)

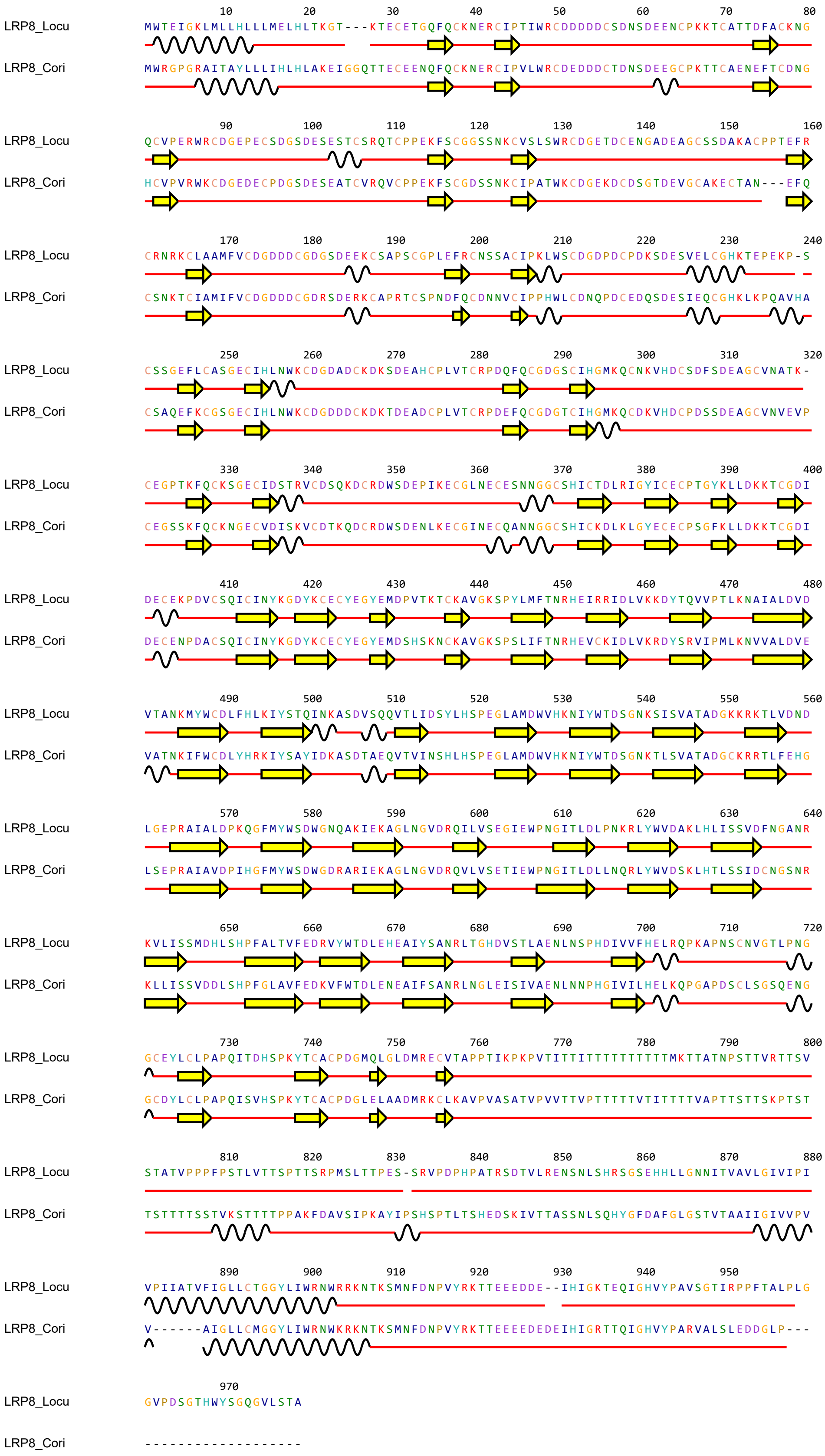

LEGEND
- characters: primary amino acid sequence
- red lines: non-structured traits of the amino acid sequence
- black colored waiving lines: alpha helices
- yellow-colored arrows: beta sheets

download svg file : here

previous

External Links

Cite 2dSS

If you use 2dSS, please cite:.

Contact Us

For questions, comments, or suggestions feel free to contact us.

Click here

Copyright © LCQB. All Rights Reserved

Design by J. Bernardes inspired in DreamTemplate

2dSS secondary structure visualization

View 2d Alignment

Comparison between C. orientalis (Cori) and Takifugu rubripes XP011613412.2

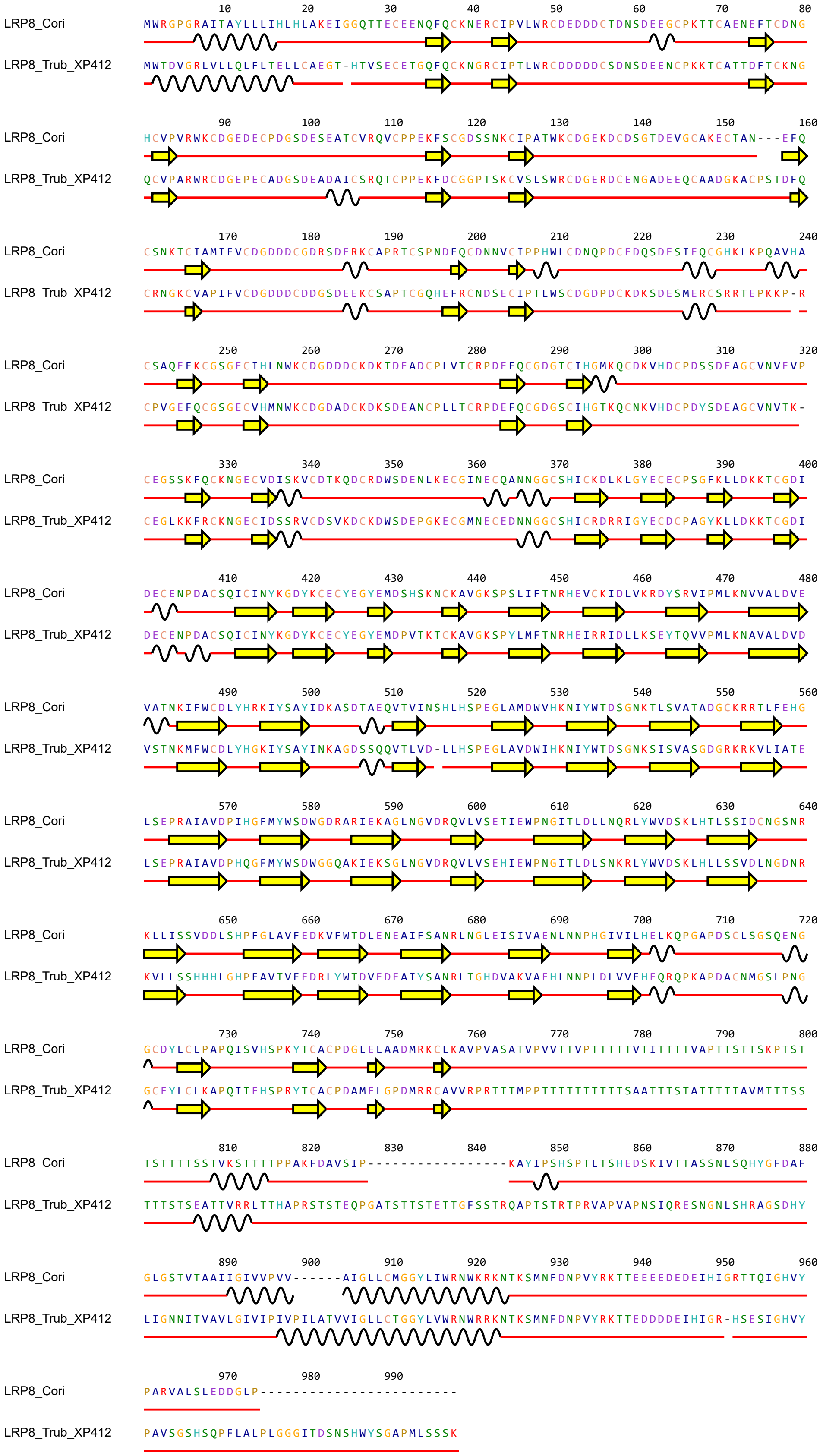

LEGEND
- characters: primary amino acid sequence
- red lines: non-structured traits of the amino acid sequence
- black-colored waiving lines: alpha helices
- yellow-colored arrows: beta sheets

download svg file : here

previous

External Links

Cite 2dSS

If you use 2dSS, please cite:.

Contact Us

For questions, comments, or suggestions feel free to contact us.

Click here

Copyright © LCQB. All Rights Reserved

Design by J. Bernardes inspired in DreamTemplate

2dSS secondary structure visualization

View 2d Alignment

Comparison between C. orientalis (Cori) and Takifugu rubripes XP011614898.2

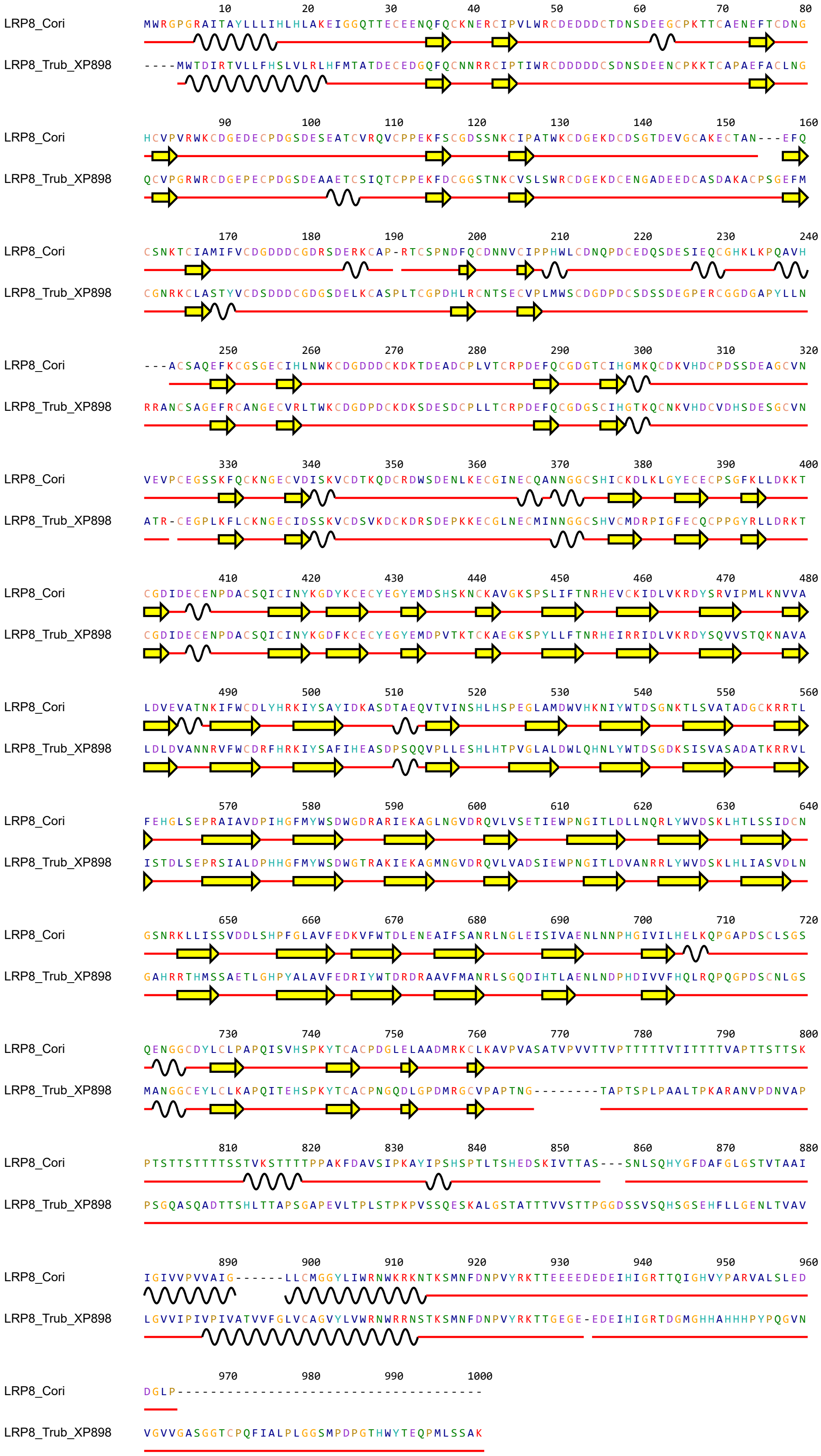

LEGEND
- characters: primary amino acid sequence
- red lines: non-structured traits of the amino acid sequence
- black-colored waiving lines: alpha helices
- yellow-colored arrows: beta sheets

download svg file : here

previous

External Links

Cite 2dSS

If you use 2dSS, please cite:.

Contact Us

For questions, comments, or suggestions feel free to contact us.

Click here

Copyright © LCQB. All Rights Reserved

Design by J. Bernardes inspired in DreamTemplate

2dSS secondary structure visualization

Comparison between C. orientalis (Cori) and Pleurodeles waltl (Pwal)

View 2d Alignment

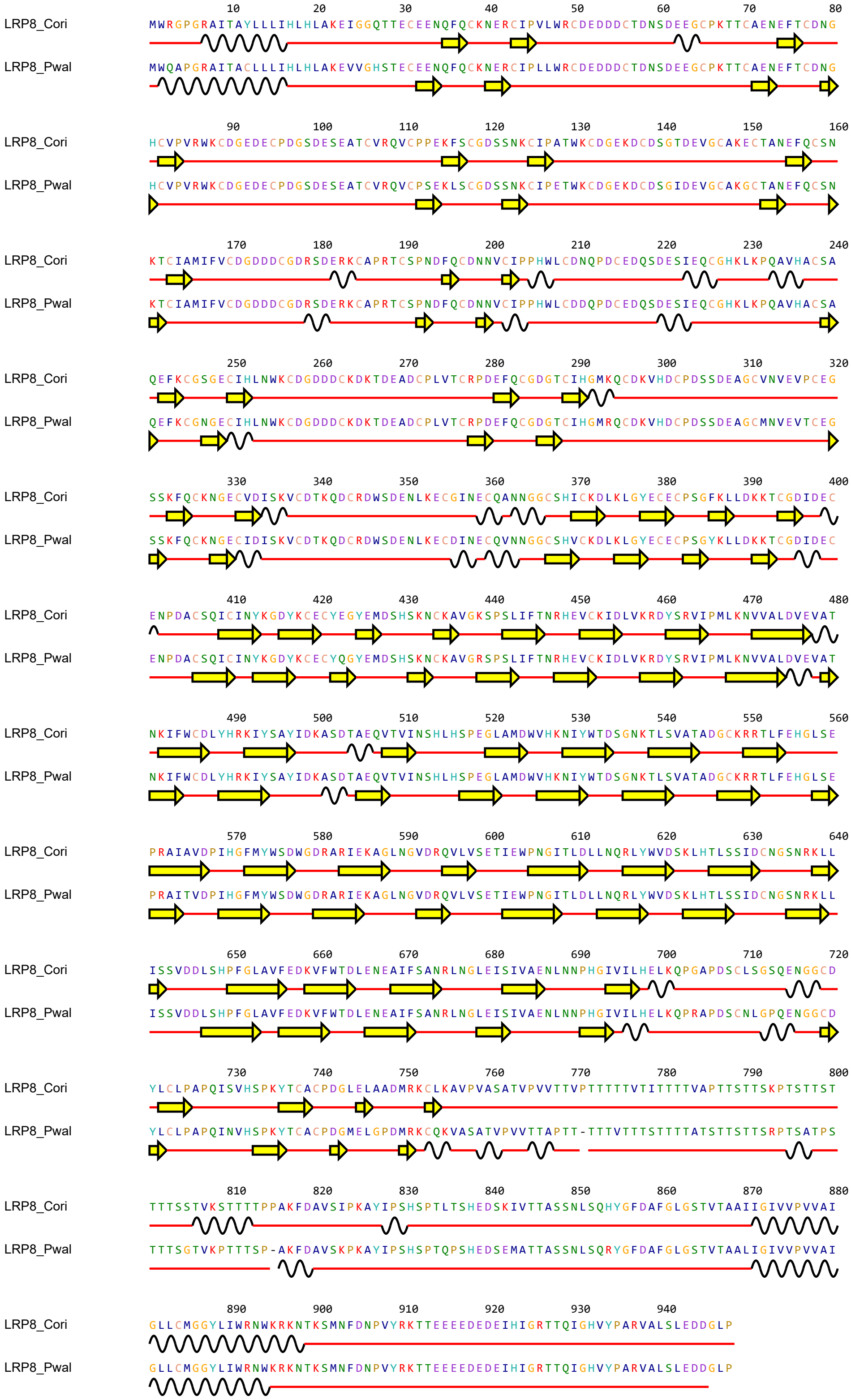

LEGEND
- characters: primary amino acid sequence
- red lines: non-structured traits of the amino acid sequence
- black-colored waiving lines: alpha helices
- yellow-colored arrows: beta sheets

download svg file : here

previous

External Links

Cite 2dSS

If you use 2dSS, please cite:.

Contact Us

For questions, comments, or suggestions feel free to contact us.

Click here

Copyright © LCQB. All Rights Reserved

Design by J. Bernardes inspired in DreamTemplate

2dSS secondary structure visualization

Comparison between C. orientalis (Cori) and Xenopus tropicalis (Xtro)

View 2d Alignment

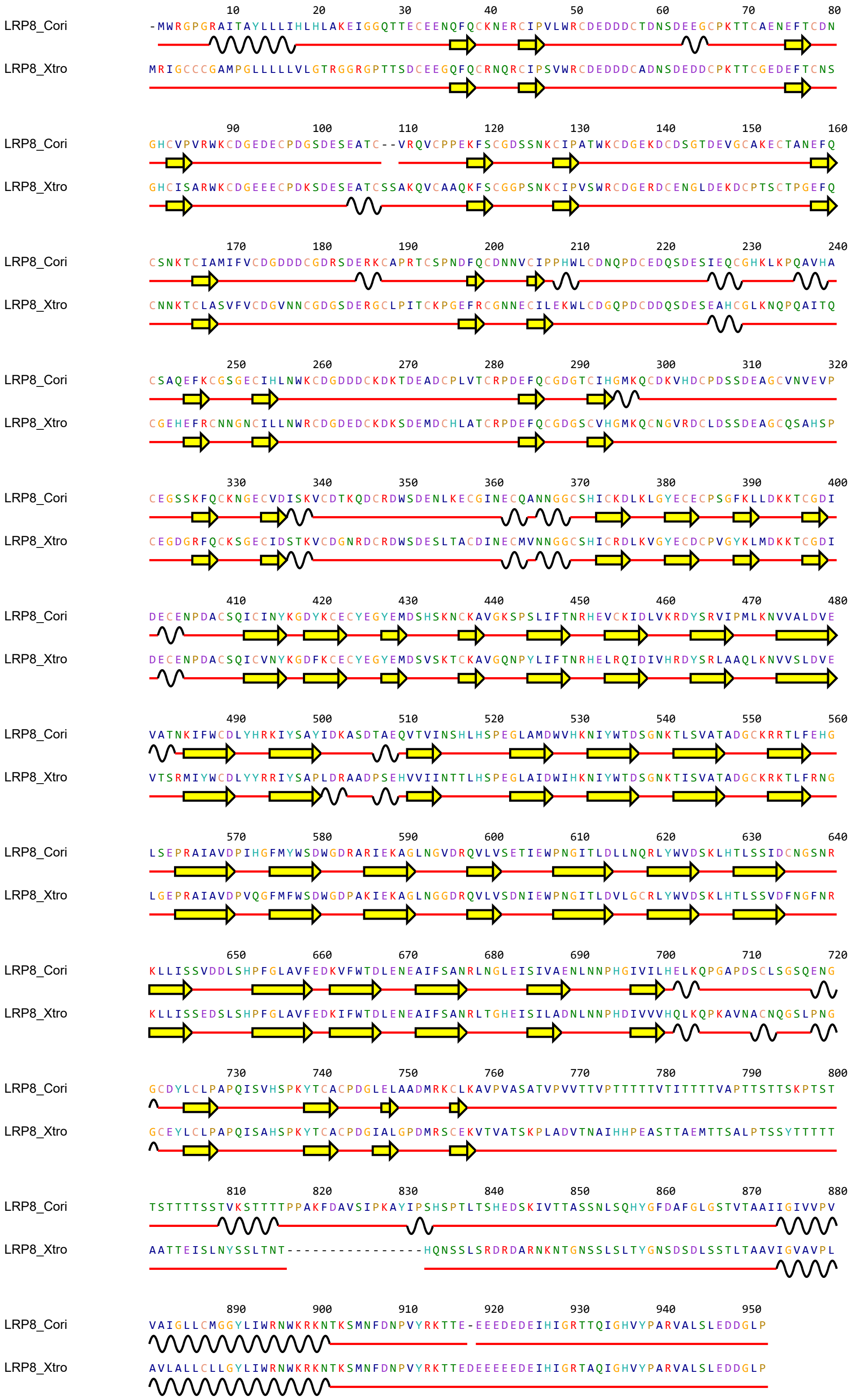

LEGEND
- characters: primary amino acid sequence
- red lines: non-structured traits of the amino acid sequence
- black-colored waiving lines: alpha helices
- yellow-colored arrows: beta sheets

download svg file : here

previous

External Links

Cite 2dSS

If you use 2dSS, please cite:.

Contact Us

For questions, comments, or suggestions feel free to contact us.

Click here

Copyright © LCQB. All Rights Reserved

Design by J. Bernardes inspired in DreamTemplate

HOME HELP EXAMPLES CONTACT US

2dSS secondary structure visualization

Comparison between C. orientalis (Cori) and Pelodiscus sinensis (Psin)

View 2d Alignment

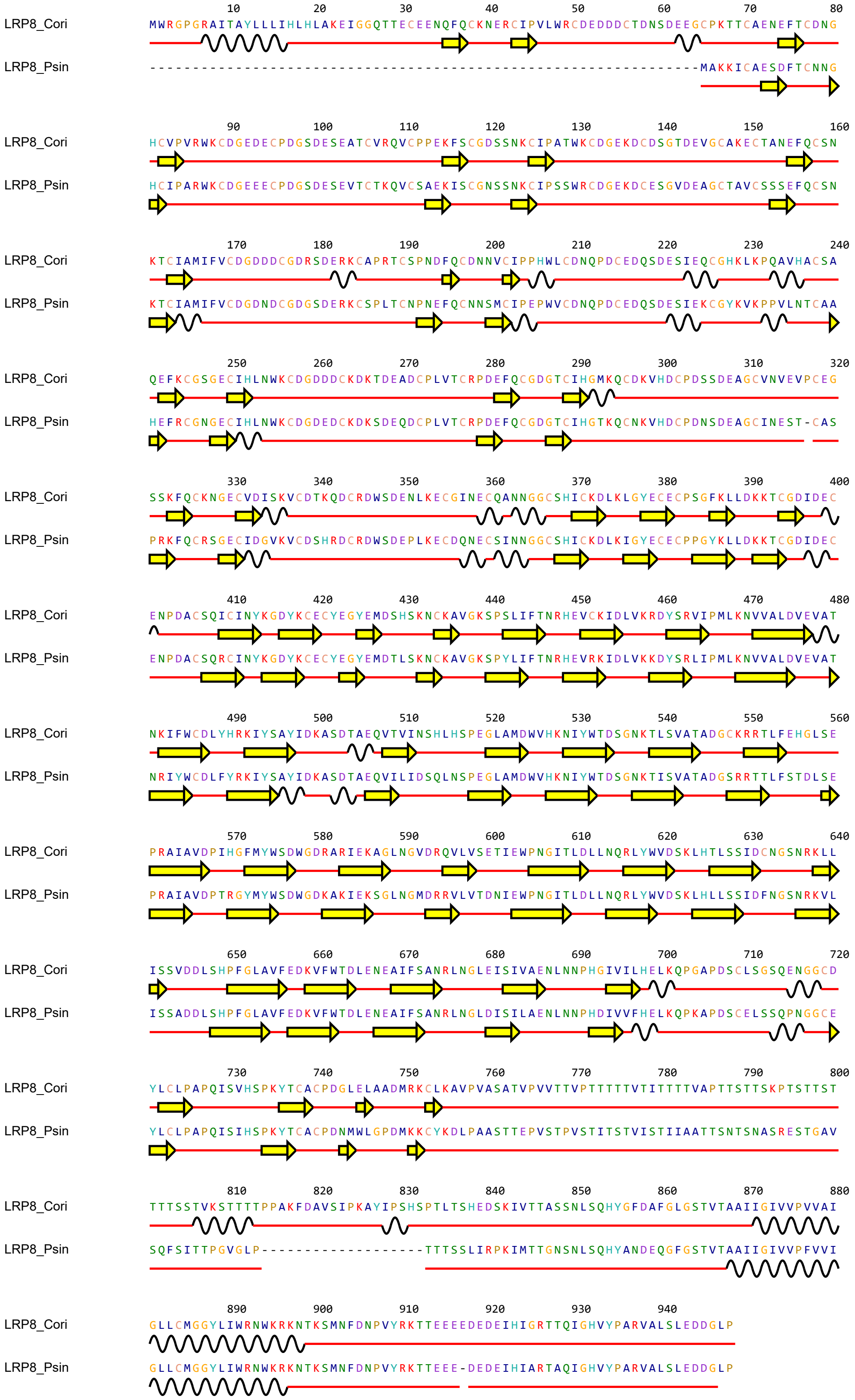

LEGEND

- characters: primary amino acid sequence
- red lines: non-structured traits of the amino acid sequence
- black-colored waiving lines: alpha helices
- yellow-colored arrows: beta sheets

download svg file : here

previous

External Links

Cite 2dSS

If you use 2dSS, please cite:.

Contact Us

For questions, comments, or suggestions feel free to contact us.

Click here

Copyright © LCQB. All Rights Reserved

Design by J. Bernardes inspired in DreamTemplate

HOME HELP EXAMPLES CONTACT US

2dSS

secondary structure visualization

Comparison between C. orientalis (Cori) and Gallus gallus (Ggal)

View 2d Alignment

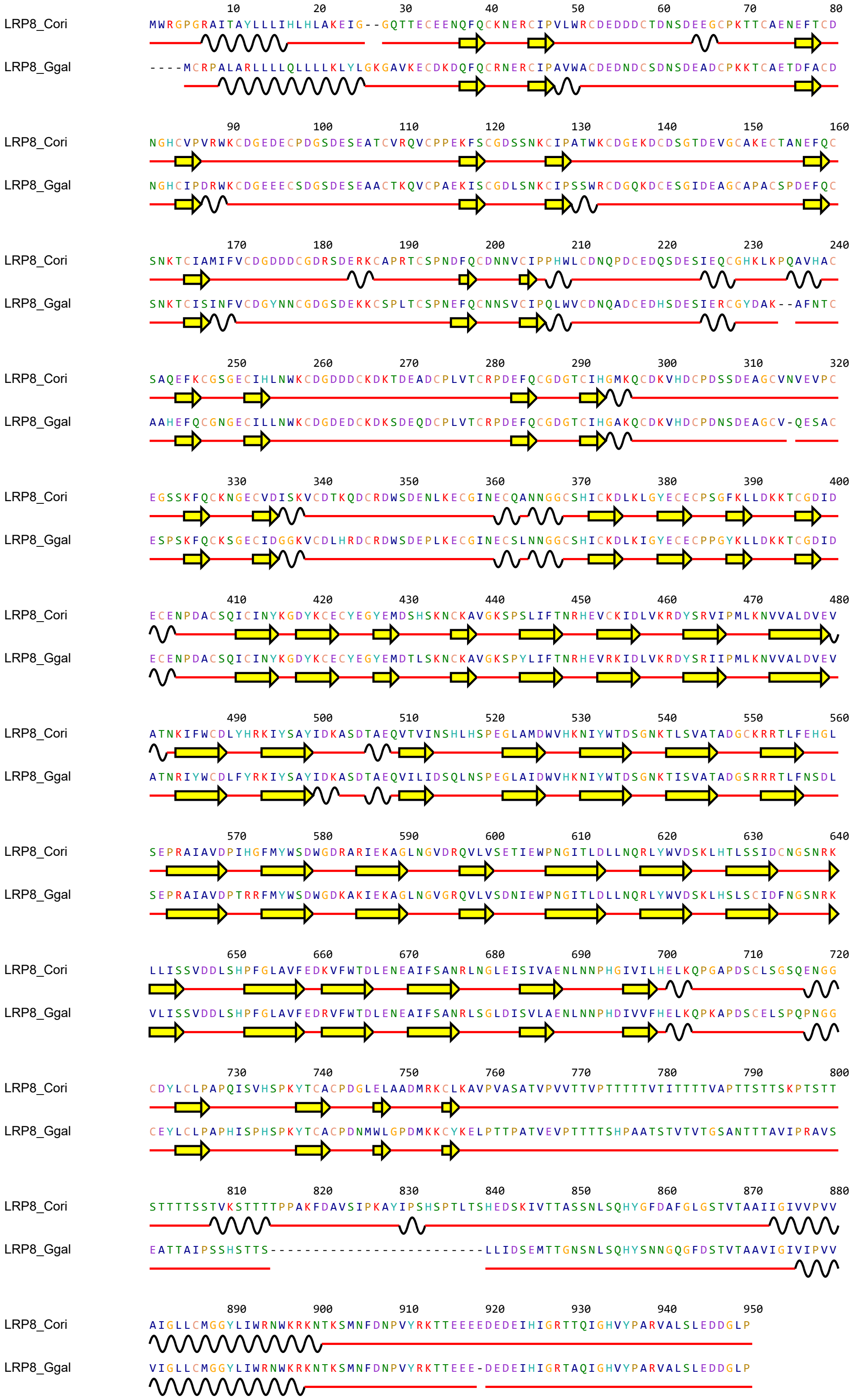

LEGEND

- characters: primary amino acid sequence
- red lines: non-structured traits of the amino acid sequence
- black-colored waiving lines: alpha helices
- yellow-colored arrows: beta sheets

download svg file : here

previous

External Links

Cite 2dSS

If you use 2dSS, please cite:.

Contact Us

For questions, comments, or suggestions feel free to contact us.

Click here

Copyright © LCQB. All Rights Reserved

Design by J. Bernardes inspired in DreamTemplate

2dSS secondary structure visualization

View 2d Alignment

Comparison between C. orientalis (Cori) and Mus musculus (Mmu)

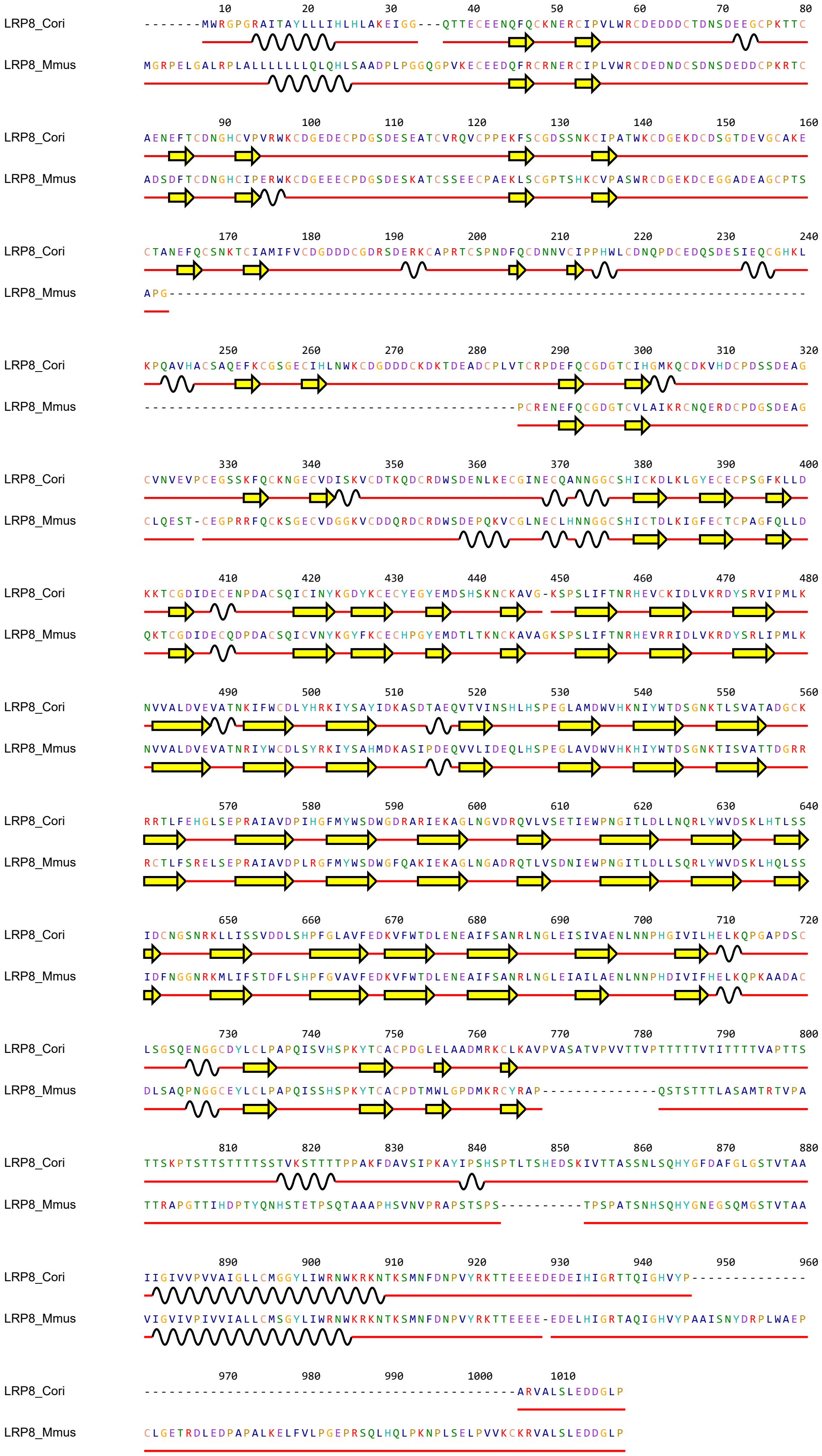

LEGEND
- characters: primary amino acid sequence
- red lines: non-structured traits of the amino acid sequence
- black-colored waiving lines: alpha helices
- yellow-colored arrows: beta sheets

download svg file : here

previous

External Links

Cite 2dSS

If you use 2dSS, please cite:.

Contact Us

For questions, comments, or suggestions feel free to contact us.

Click here

Copyright © LCQB. All Rights Reserved

Design by J. Bernardes inspired in DreamTemplate

2dSS secondary structure visualization

View 2d Alignment

Comparison between C. orientalis (Cori) and Homo sapiens (Hsap)

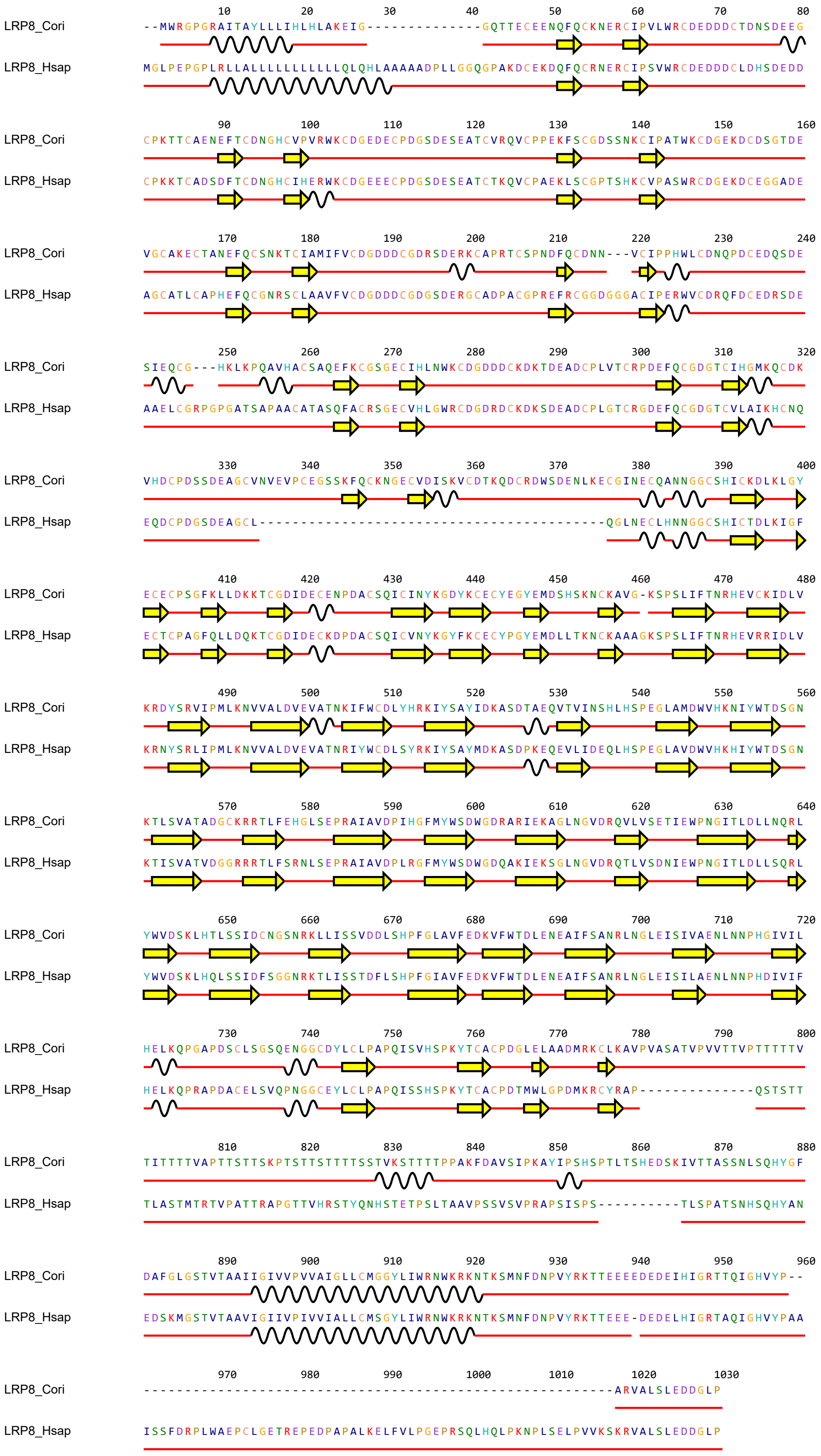

LEGEND
- characters: primary amino acid sequence
- red lines: non-structured traits of the amino acid sequence
- black-colored waiving lines: alpha helices
- yellow-colored arrows: beta sheets

download svg file : here

previous

External Links

Cite 2dSS

If you use 2dSS, please cite:.

Contact Us

For questions, comments, or suggestions feel free to contact us.

Click here

Copyright © LCQB. All Rights Reserved

Design by J. Bernardes inspired in DreamTemplate
